# *In silico*-guided discovery and in vitro validation of novel sugar-tethered lysinated carbon nanotubes for targeted drug delivery of doxorubicin

**DOI:** 10.1007/s00894-024-06061-5

**Published:** 2024-07-10

**Authors:** Chanchal Kiran Thakur, Fábio G. Martins, Chandrabose Karthikeyan, Subhasmita Bhal, Chanakya Nath Kundu, N. S. Hari Narayana Moorthy, Sérgio F. Sousa

**Affiliations:** 1 Cancept Therapeutics Laboratory, Department of Pharmacy, Indira Gandhi National Tribal University, Lalpur, Amarkantak, Anuppur, Madhya Pradesh 484887 India; 2 LAQV/REQUIMTE, BioSIM-Departamento de Biomedicina, Faculdade de Medicina, Universidade Do Porto, 4200-319 Porto, Portugal; 3 Cancer Biology Division, School of Biotechnology, KIIT Deemed to Be University, Campus-11, Patia,, Bhubaneswar, Odisha 751024 India

**Keywords:** Multiwalled carbon nanotubes, Breast cancer, Drug delivery, Anti-cancer drug, Molecular dynamics simulations

## Abstract

**Context:**

Multiwalled carbon nanotubes (MWCNTs) functionalized with lysine via 1,3-dipolar cycloaddition and conjugated to galactose or mannose are potential nanocarriers that can effectively bind to the lectin receptor in MDA-MB-231 or MCF-7 breast cancer cells. In this work, a method based on molecular dynamics (MD) simulation was used to predict the interaction of these functionalized MWCNTs with doxorubicin and obtain structural evidence that allows a better understanding of the drug loading and release process. The MD simulations showed that while doxorubicin only interacted with pristine MWCNTs through π-π stacking interactions, functionalized MWCNTs were also able to establish hydrogen bonds, suggesting that the functionalized groups improve doxorubicin loading. Moreover, the elevated adsorption levels observed for functionalized nanotubes further support this enhancement in loading efficiency. MD simulations also shed light on the intratumoral pH-specific release of doxorubicin from functionalized MWCNTs, which is induced by protonation of the daunosamine moiety. The simulations show that this change in protonation leads to a lower absorption of doxorubicin to the MWCNTs. The MD studies were then experimentally validated, where functionalized MWCNTs showed improved dispersion in aqueous medium compared to pristine MWCNTs and, in agreement with the computational predictions, increased drug loading capacity. Doxorubicin-loaded functionalized MWCNTs demonstrated specific release of doxorubicin in tumor microenvironment (pH = 5.0) with negligible release in the physiological pH (pH = 7.4). Furthermore, doxorubicin-free MWNCT nanoformulations exhibited insignificant cytotoxicity. The experimental studies yielded nearly identical results to the MD studies, underlining the usefulness of the method. Our functionalized MWCNTs represent promising non-toxic nanoplatforms with enhanced aqueous dispersibility and the potential for conjugation with ligands for targeted delivery of anti-cancer drugs to breast cancer cells.

**Methods:**

The computational model of a pristine carbon nanotube was created with the buildCstruct 1.2 Python script. The lysinated functionalized groups were added with PyMOL and VMD. The carbon nanotubes and doxorubicin molecules were parameterized using the general AMBER force field, and RESP charges were determined using Gaussian 09. Molecular dynamics simulations were carried out with the AMBER 20 software package. Adsorption levels were calculated using the water-shell function of cpptraj. Cytotoxicity was evaluated via a MTT assay using MDA-MB-231 and MCF-7 breast cancer cells. Drug uptake of doxorubicin and doxorubicin-loaded MWCNTs was measured by fluorescence microscopy.

**Supplementary Information:**

The online version contains supplementary material available at 10.1007/s00894-024-06061-5.

## Introduction

Nanocarriers have a long history that may be traced back to Nobel Laureate Sir Paul Ehrlich [1]. Nanocarriers have been the subject of intense research over the past few decades due to their enormous potential in drug delivery. Carbon-based, lipid-based, inorganic-based, and carbohydrate-based nanocarriers are among the several types of nanocarriers that have been developed [2, 3]. Carbon nanotubes (single-walled and multiwalled) constitute a novel class of nanoplatforms with diverse applications, such as drug delivery, diagnostics, and biosensing [4–6].

The use of multiwalled carbon nanotubes (MWCNTs) in the biomedical field has increased substantially in recent years due to their unique properties, such as increased surface area, nanoform, and ease of functionalization with diverse groups. MWCNTs have additional advantages over single-walled carbon nanotubes (SWCNTs), including a high loading capacity, sustained drug release due to their multilayered structure, and the potential to be coupled with suitable ligands for targeted drug delivery [7–10]. MWCNTs are potentially attractive nanocarriers for cancer chemotherapeutics, as several chemotherapeutic drugs can be linked covalently or non-covalently to the surface of the nanotubes [11–15]. Despite these benefits, pristine MWCNTs tend to aggregate due to their hydrophobicity, making them difficult to disperse or dissolve in aqueous or organic solvents and potentially limiting their application. Therefore, the surface of MWCNTs must be appropriately functionalized to improve their aqueous dispersibility and make them potentially viable for biological applications. Multiple functionalization strategies for MWCNTs have been described in the literature [12, 16]. Functionalization of MWCNTs to increase solvent dispersibility and provide anchoring groups for covalently linking ligands for targeted drug delivery typically requires hazardous chemicals and expensive reagents such as chitosan, polyethylene glycol, ethylene diamine, peptides, vitamins, and antibodies [17–19]. MWCNTs are often functionalized by introducing carboxyl groups, amino groups, or hydroxyl groups on its surface, which are then conjugated to a targeting ligand *via* a linker or through direct conjugation. MWCNTs are carboxylated with sulfuric acid, nitric acid, or a combination of the two, whereas acylation and hydroxylation are accomplished with thionyl chloride and hydrogen peroxide, respectively [20]. Poly (ethylene glycol) bis(amine) and ethylenediamine are the most often employed linkers for amino functionalization and enhancing the dispersibility of MWCNTs. However, PEG-bis-amine is expensive and ethylenediamine’s toxicity limits its use as a linker. Consequently, a biocompatible, biodegradable, non-toxic, and less expensive linker is required for amino functionalization and MWCNT dispersibility enhancement.

**Fig. 1:**
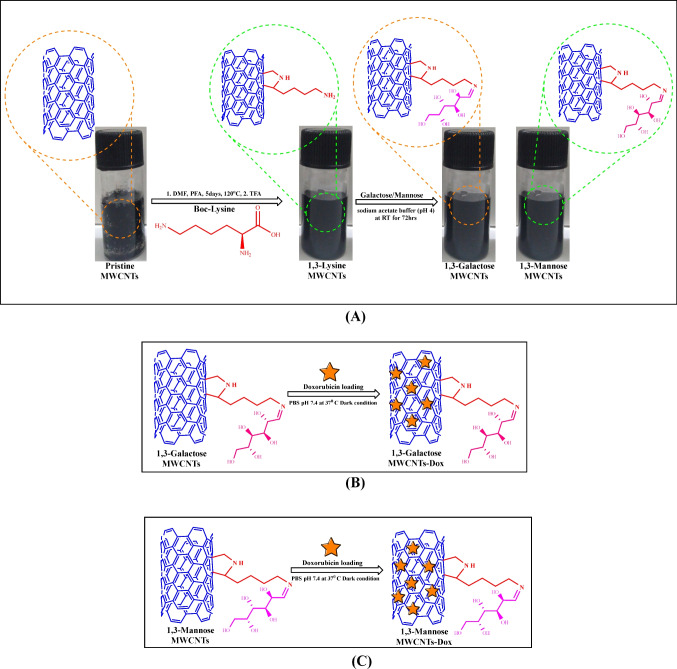
Schematic representation of synthesis. **A** 1,3 dipolar cycloaddition of lysine and covalent conjugation of ligands (galactose/mannose) in pristine MWCNTs. **B** Dox loaded into galactose-modified 1,3-lysine-MWCNTs. **C** Dox loaded into mannose-modified 1,3-lysine-MWCNTs

Lysine is a biocompatible, biodegradable, non-toxic, and inexpensive essential amino acid that can be used to functionalize MWCNTs [21–23]. Few studies have described the conjugation of lysine to carbon nanotubes to improve their dispersion and impart antibacterial properties [24]. Zardini et al. reported the development of lysine and arginine conjugated carboxylated MWCNTs with antibacterial properties, as well as improved aqueous dispersibility [25]. Amiri et al. functionalized MWCNTs with lysine by diazonium reaction to increase the aqueous solubility of MWCNTs and their antibacterial activity [26]. Both of the documented methods use expensive and non-scalable microwave heating as well as hazardous chemicals (Conc. H_2_SO_4_, Conc. HNO_3_, azo compounds) for lysine functionalization of MWCNTs. In light of these findings, the current research intends to covalently link lysine with MWCNTs *via* a 1,3-dipolar cycloaddition strategy, which would eliminate the requirement for hazardous chemicals and reduce the number of steps typically required for such a process. In addition, 1,3-dipolar cycloaddition procedure used to link lysine to MWCNTs (Fig. 1) provides a free ε-amino group that can be coupled to ligands for targeted drug delivery. Carbohydrate ligands, such as galactose and mannose, which are biocompatible and affordable, were attached to lysine functionalized MWCNTs to facilitate cancer targeting *via* the lectin receptor, which is abundantly expressed in malignant cells [27–30]. Doxorubicin (Dox) has been reported as an anti-cancer agent in metastatic breast cancer and triggers apoptosis by DNA intercalation, topoisomerase-II inhibition, etc. However, this drug has some drawbacks which are drug efflux and chemoresistance exerted by cancer stem cells present among cancer cells. Thus, poor solubility, lack of selectivity, and off-target activity of Dox cause several side effects, including cardiotoxicity, acute nausea, and vomiting, restricting it to clinical trials [31]. Therefore, the overarching objective is to develop environmentally benign and economically viable MWCNT-based nanocarriers with enhanced dispersibility and targeting efficacy that can efficiently deliver doxorubicin to breast cancer cells.

Molecular dynamics (MD) is a powerful technique that offers quantitative and qualitative data on molecules, including information about interactions and structural data. MD simulations provide atomic structural information that is difficult to obtain in experimental research [32, 33]. This technique has become widely employed in order to understand the impact of functionalized groups, molecular interactions, and the effects of pH changes in drug delivery carriers [34, 35]. The loading, arrangement, and release of Dox on lysine-MWCNTs, galactose-MWCNTs, and mannose-MWCNTs were investigated in the current work using molecular dynamics simulations at two distinct pH levels (neutral and acidic). By providing structural, atomic-level evidence, these studies facilitate a deeper understanding of experimental results.

## Experimental section

### Materials

Doxorubicin (Dox) was supplied as a gift sample from M/s Cipla (P) Ltd. Pristine multiwalled carbon nanotube, Boc-lysine, and potassium dihydrogen phosphate were purchased from Sisco Research Laboratories, Mumbai, India. Disodium hydrogen phosphate, paraformaldehyde (PFA), galactose, and mannose were procured from M/s Central Drug House (P) Ltd., India. Dimethylformamide (DMF) and trifluoroacetic acid (TFA) were supplied by M/s Molychem (P) Ltd., India. Sodium acetate and sodium hydroxide were procured from M/s Qualikema Laboratory Reagents, India. Sodium chloride and glacial acetic acid were purchased from Loba-Chemie (P) Ltd., India.

### Computational methods

#### Model preparation

For this study, three different functionalized MWCNT models were built: (1) lysine (1,3-LyMW), (2) lysine bound to mannose (1,3-MAMW), and finally (3) lysine bound to galactose (1,3-GAMW).

In order to build each system, the first step was to generate a dual-layer, pristine carbon nanotube with the buildCstruct 1.2 Python script [36]. The inner and outer layers of the nanotube presented an armchair structure and a total length of 201.0 Å. Diameters of 95.4 Å and 102.2 Å were used for the inner and outer layers of the nanotube.

For each model, the outer wall of the nanotube was functionalized with a total of 195 lysine-carbohydrate groups. For this, PyMOL and VMD were used [37, 38]. The last step was to add 100 Dox molecules to the system using packmol [39]. These molecules were placed at random positions within a distance of 10 to 40 Å to the nanotube.

To study the pH-dependent loading and unloading of Dox, it was necessary to create a version of the system that represented neutral pH conditions and another with acidic pH. According to the literature, there would be a mixture of doxorubicin molecules with protonated and deprotonated amine groups in both acidic and neutral conditions [40, 41]. In this study, in order to better compare the behavior of Dox in different pH conditions, the amine group was deprotonated in neutral conditions, while in acidic conditions, the amine group was protonated. Despite the mixture of protonation states observed during neutral conditions, multiple MD simulation studies have used deprotonated Dox in simulations with neutral pH, and by comparing the extreme scenarios, the simulations provide a clearer demonstration of the impact of pH in the absorption of Dox molecules to the nanotube [34, 42–47].

The carbon nanotubes and Dox molecules were parameterized with Antechamber [48] using the general AMBER force field (GAFF) [49], and RESP charges were determined using Gaussian 09 [50] with the at HF/6-31G(d) basis set. Additionally, Antechamber’s residuegen was used to parameterize the lysine, mannose, and galactose functionalized groups.

#### Molecular dynamics simulation study of lysine and ligands (galactose/mannose) functionalized MWCNTs

MD simulations were performed with the AMBER 20 software suite [48]. The systems were prepared using the LEAP toolkit. Each system was placed in a TIP3P water box, with a minimum distance of 12 Å between the system and the side of the box. After the addition of the water molecules, all systems consisted of over 800,000 atoms. A cut-off distance of 10.0 Å was used for the electrostatic and Lennard-Jones interactions, and the particle mesh Ewald summation method was used to calculate long-range electrostatic interaction. The SHAKE algorithm was used to constrain bonds involving hydrogen atoms.

Each model underwent four consecutive minimization stages. In each of the four stages, the minimization process was applied to the following atoms: step 1—water molecules (2500 steps), step 2—hydrogen atoms (2500 steps), step 3—inner wall and doxorubicin molecules (2500 steps), step 4—complete system optimization (10,000 steps). For each step, the first half was run using the steepest descent algorithm, while the second half used the conjugate gradient algorithm. After all minimization steps, the systems were subjected to a 50 ps MD equilibration procedure where each system was progressively heated to 310.15 K using the Langevin thermostat. This step was performed at constant volume (canonical thermodynamic ensemble (NVT)). Using the same ensemble and temperature, the systems were equilibrated for an additional 50 ps. Finally, the production phase was run with constant pressure (isothermal–isobaric ensemble (NPT)), with a pressure of 1 bar (Berendsen barostat) and with the temperature maintained at 310.15 K. Each production phase had a length of 300 ns.

#### Simulation analysis

Root mean square deviation (RMSD) and the MD simulations’ general properties were used to evaluate the stability and convergence of each system. The cpptraj tool from AMBER and the VMD molecular visualization program were used to analyze the trajectories.

The adsorption of Dox to the MWCNTs was estimated with the water-shell command of cpptraj. As was described in our previous work [32], this command calculates the number of water molecules surrounding each Dox molecule. The radial distribution function (RDF) in cpptraj was utilized to ascertain the number of water molecules within the first solvation sphere surrounding a free Dox molecule. Analysis revealed that the first solvation sphere contained a range of 23 to 37 water molecules. To classify a Dox molecule as adsorbed onto the MWCNT, its water-shell must contain fewer water molecules than the minimum number determined in the RDF calculations, specifically 23 or fewer water molecules. Additionally, the Molecular Mechanics Generalized Born Surface Area (MM-GBSA) method was used to estimate the binding free energy between one Dox molecule per system and the MWCNTs. In order to achieve this, the MMPBSA.py script available in AMBER was used [51]. The calculations considered the last 200 ns of the MD simulation of every system, with a total of 400 frames and an ionic strength of 0.150 mol dm^−3^.

### Experimental validation

#### Dispersion stability study

PMW and modified MWCNTs (1,3-LyMW, 1,3-GAMW, and 1,3-MAMW) were dispersed in deionized water for 30 min by ultrasonication, and the dispersion was visually analyzed for up to 30 days [12, 52].

#### Doxorubicin loading

Twenty milligrams of PMW and modified MWCNTs (1,3-LyMW, 1,3-GAMW, and 1,3-MAMW) were suspended in 20 ml of Dox solution (1 mg/ml) and stirred at 37 °C in the dark for 24 h. After completion of the reaction, the unbound or free Dox was removed from the suspension by repetitive centrifugation (12,000 rpm at 4 °C for 15 min) and redispersion with PBS (pH 7.4) until the supernatants become colorless and no UV absorbance at 481 nm was observed (UV Spectrophotometer 1900i, Shimadzu, Tokyo, Japan) [53–56]. The Dox loading percentage was determined in triplicate, and the Dox loading percentage efficiency was computed using the following formula.

$$\mathrm{Drug}\;\mathrm{loading}\;\mathrm{efficiency}\;(\%)=\frac{\mathrm{WID}-\mathrm{WFD}}{\mathrm{WID}}\times100$$

where WID is weight of initial loaded Dox and WFD is weight of unbounded/free Dox.

#### Drug release mechanism

Drug release from Dox-loaded modified MWCNTs (1,3-LyMW-Dox, 1,3-GAMW-Dox, and 1,3-MAMW-Dox) was investigated in phosphate buffer (pH 7.4) and acetate buffer (pH 5.0) at 37 °C as per the protocols reported previously [56–58]. Dox-loaded modified MWCNTs (1 mg/ml) were dispersed in the medium and placed in a dialysis bag that was suspended in release media (80 ml). The sink temperature was maintained at 37 °C with constant stirring. Five-milliliter aliquots of each sample solution were taken at predefined time intervals (varying from 0.5 to 120 h) and replaced with an equal volume of new diffusion medium to maintain the sink condition [54]. The concentration of Dox released was measured using a UV spectrophotometer set at 481 nm, and the cumulative fraction of drug release versus time was calculated using the following formula:

$$\mathrm{Drug}\;\mathrm{release}\;(\%)=\frac{\mathrm{Drug}\;\mathrm{amount}\;\mathrm{rreleased}\;\mathrm{at}\;\mathrm{time}}{\mathrm{Initial}\;\mathrm{drug}\;\mathrm{amount}\;\mathrm{loaded}\;\mathrm{on}\;\mathrm{MWCNT}\;\mathrm{formulations}}\times100$$

#### Cell culture and reagents

MDA-MB-231 and MCF-7 breast cancer cells were cultured in RPMI-1640 (50:50, v/v) and DMEM (50:50, v/v) media, respectively, which were supplemented with 1% antibiotic (10 mg/ml of streptomycin and 100 U/ml of penicillin in 0.9% normal saline), 10% fetal bovine serum (FBS), and 1.5 mM L-glutamine at 37 °C with 5% CO_2_ in a relative humidified atmosphere (at 37 °C). These cell culture reagents were bought from HiMedia, India. The cells were cultivated as a monolayer of adherent cells [59].

#### Cell cytotoxicity assay

To check the cytotoxic potentiality, MTT [3-(4,5-dimethylthiazol-2yl-)-2,5-diphenyltetrazolium bromide] assay was carried out following the standard protocol [58–61]. The cells were seeded at a density of 8000–10,000 cells per well in triplicate in a 96-well tissue culture plate and incubated for 24 h. After 60–70% confluency, the cells were treated with the test substances (including 0.78–50 μg/ml of plain Dox, 1,3-GAMW-Dox, 1,3-MAMW-Dox, 1,3-GAMW, and 1,3-MAMW). Following 48 h of incubation, media was removed and 100 μl of MTT reagent solution (0.05%) was added in each well and incubated overnight at 37 °C for the formation of formazan crystals. Then, the media was removed, and 100 μl/well of 0.2% NP-40 detergent solution was added and incubated for 2 h in dark to dissolve the formazan crystals [62]. The absorbance was then measured at 570 nm in a spectrophotometer using a microplate reader (Berthold, Germany), and the percentage of cell viability was calculated and plotted against concentrations of the drugs.

#### Cell uptake study by fluorescence microscopy

Doxorubicin has the property of emitting red fluorescence when excited at 470 nm. In this study, drug uptake of Dox and Dox-loaded MWCNTs was measured by fluorescence microscopy. Briefly, cells were seeded at a density of 1 × 105 cells/well in a 12-well plate and incubated for 24 h. The cells were subsequently treated with IC50 concentrations of Dox and Dox-loaded MWCNTs (1,3-GAMW-Dox and 1,3-MAMW-Dox) and incubated overnight. Following which, the cells were washed with 1X PBS for 2–3 times, and subsequently, images were taken using fluorescence microscopy (Nikon, Japan) [54, 59, 60].

#### Quantification of apoptosis by Annexin-V-FITC/PI dual staining

To measure the apoptosis after the cells were treated with Dox and Dox-loaded MWCNTs (1,3-GAMW-Dox and 1,3-MAMW-Dox), Annexin-V-FITC/PI dual staining was performed using the methodology reported previously [56, 58]. Briefly, MDA-MB-231 and MCF-7 (1 × 105 cells) were seeded on to each well of 6 well plates and treated accordingly. After 48 h incubation, the cells were harvested, collected, and washed with 1X PBS, followed by staining with Annexin-V-FITC and PI stains for 10 min. Further, approximately 10,000 cells/sample were sorted by flow cytometry (FACS CANTO II, Becton & Dickinson, CA, USA). The data were analyzed using FACS Diva software [63].

#### Statistical analysis

All the experiments were performed in triplicate, and data were expressed as the mean ± SD. One-way ANOVA multiple comparison test and GraphPad Prism were used for the analysis of data. Statistical significance of differences was indicated as the *P* value, which was considered as >0.05 (non-significant (*)), <0.01 (significant (**)), and <0.001 (highly significant (***)).

## Result and discussion

### MD simulation analysis of functionalized MWCNTs

#### Interactions between Dox and MWCNTs

**Fig. 2:**
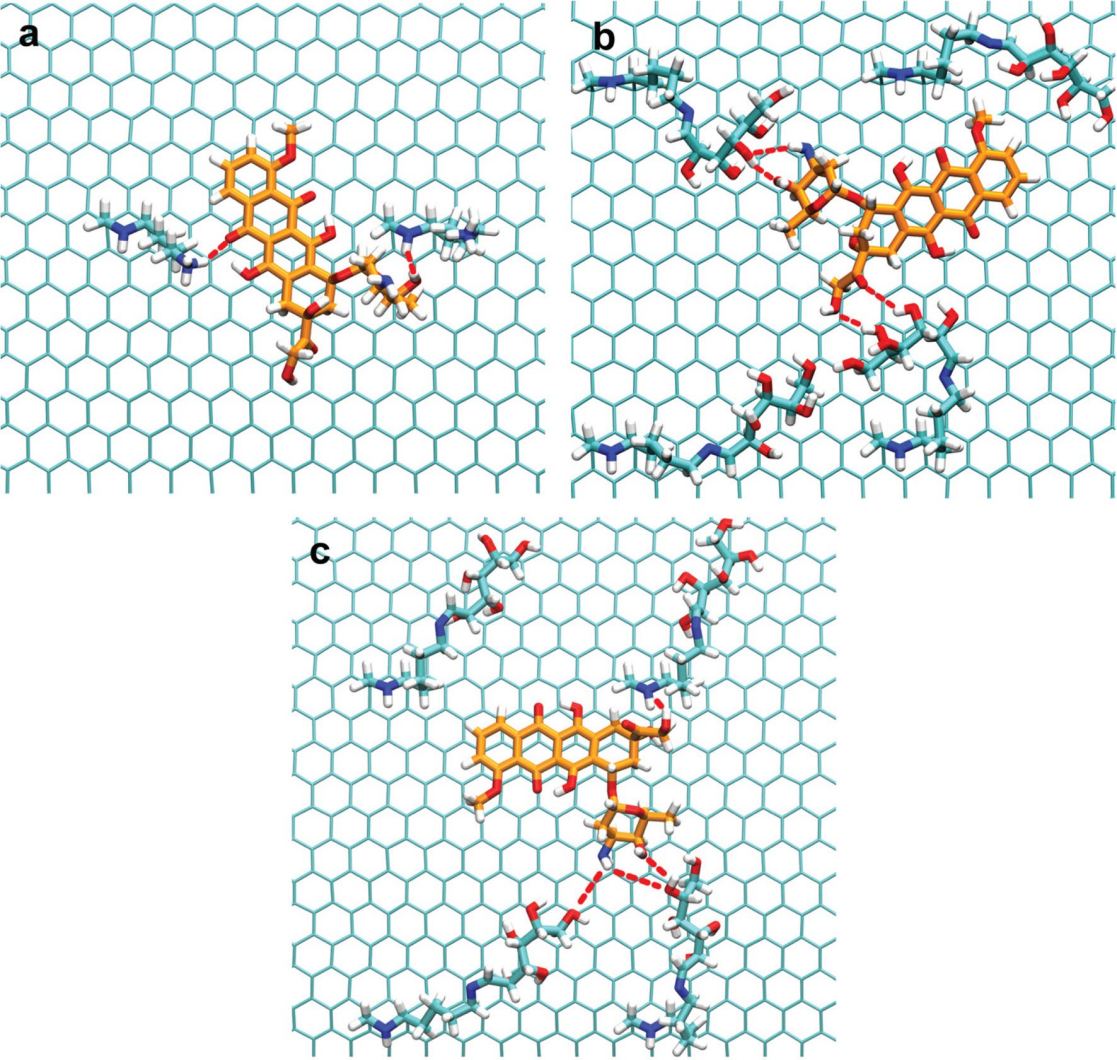
Interactions between the Dox molecules and the MWCNTs functionalized with **a** lysine (1,3-LyMW), **b** galactose (1,3-GAMW), and **c** mannose (1,3-MAMW)

Molecular dynamics (MD) simulations were used to investigate the interaction between Dox molecules and functionalized MWCNTs. As was demonstrated in our previous study [32], the functionalized groups allow the formation of hydrogen bonds between the MWCNTs and Dox molecules, in addition to the π-π stacking interactions possible with pristine MWCNTs. This is due to the hydroxyl groups from Dox being able to form hydrogen bonds with the lysine’s amine and carboxyl groups and the carbohydrate’s hydroxyl groups. Figure 2 displays the interactions between the Dox molecules and each functionalized MWCNT after 300 ns of MD simulation, where the above-described interactions can be seen.

#### Adsorption of the Dox molecules to functionalized MWCNTs

**Fig. 3:**
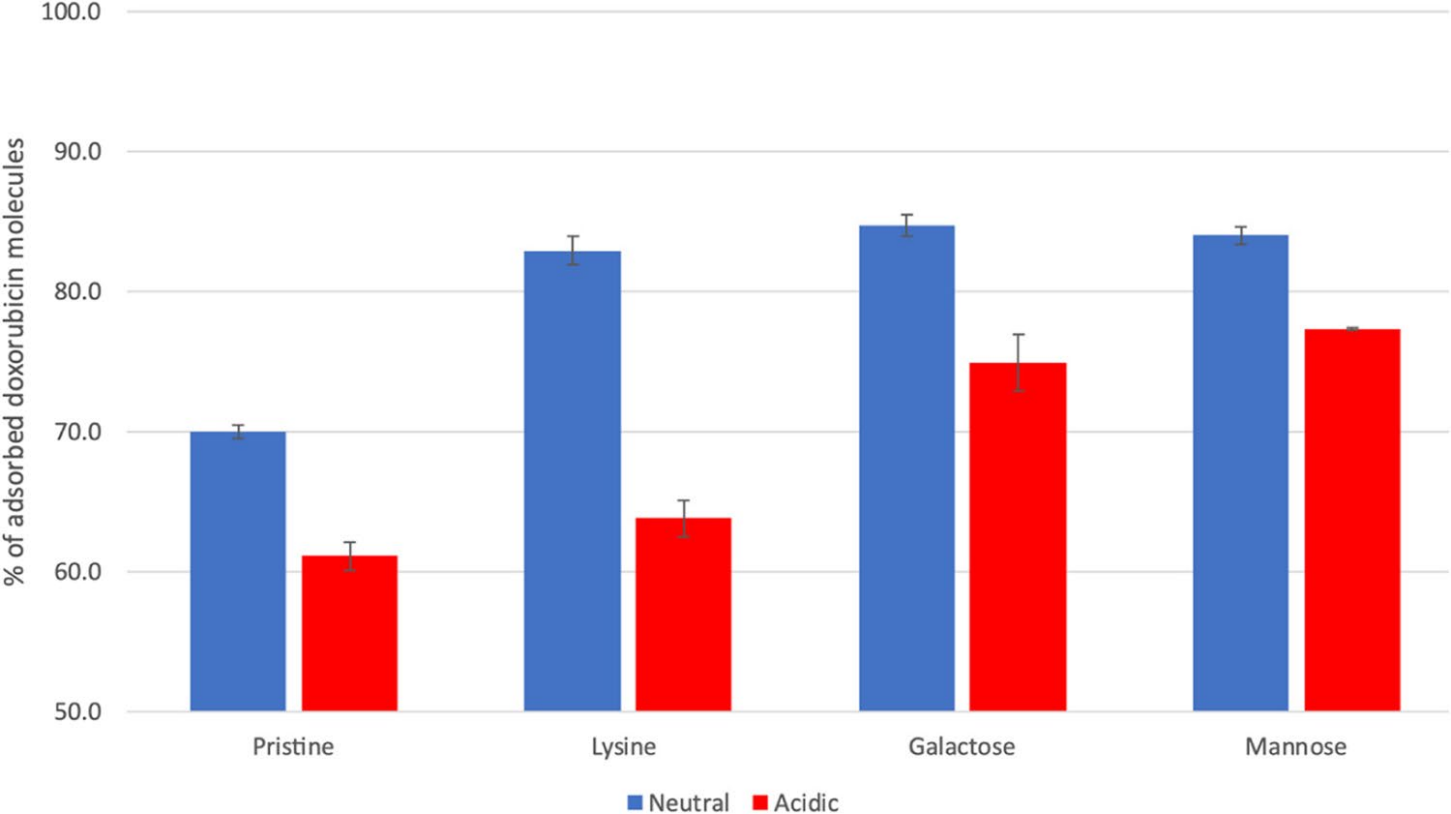
Average percentage of Dox molecules, in acidic and neutral conditions, adsorbed to pristine, lysine, galactose, and mannose functionalized MWCNTs

To determine the adsorption of the Dox molecules to the nanotubes, the water-shell command of cpptraj was used, as described on the “Computational methods” section. Each system was stabilized before the water-shell calculations were conducted to assess the proportion of adsorbed Dox molecules. Analysis of root-mean-square deviation (RMSD) suggested that all systems were stable within 150 ns of simulation. In general, all systems display a significant difference between conditions, with neutral conditions exhibiting greater levels of Dox adsorption in comparison to acidic conditions. Additionally, for comparative purposes, the results from our prior study using a pristine nanotube model are also provided [32] (Table 1 and Fig. 3).

**Table 1:** Average percentage of Dox molecules, in acidic and neutral conditions, adsorbed to 1,3-LyMW, 1,3-GAMW, and 1,3-MAMW

Average percentage of adsorbed Dox molecules		
Formulations	Neutral	Acidic
Pristine	70.0 ± 0.5	61.1 ± 1.0
Lysine (1,3-LyMW)	82.9 ± 1.0	63.8 ± 1.3
Galactose (1,3-GAMW)	84.7 ± 0.8	74.9 ± 2.0
Mannose (1,3-MAMW)	84.0 ± 0.6	77.3 ± 0.1

**Fig. 4:**
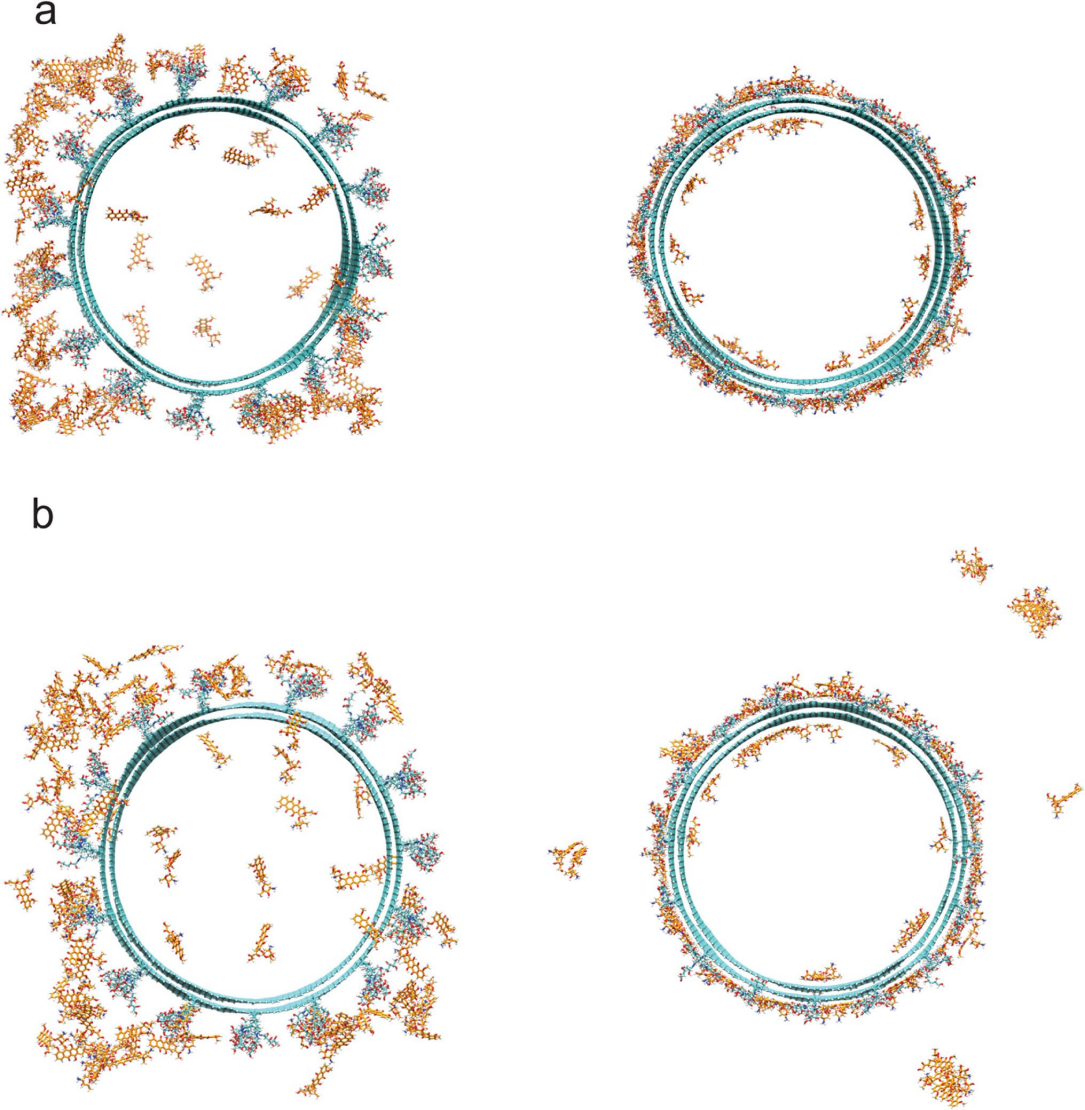
Galactose/mannose functionalized MWCNTs at the start of the simulation and at the end of the simulation under neutral conditions (**a**) and acidic conditions (**b**)

Table 1 and Fig. 3 show that incorporating functionalized groups increases the absorption of Dox, as all functionalized nanotubes demonstrate higher levels of adsorption when compared to the pristine nanotube (82.9–84.7% for functionalized MWCNTs compared to 70.0 for the pristine MWCNTs). As for the differences between neutral and acidic conditions, the most significant difference can be seen in the case of the MWCNTs functionalized with lysine (1,3-LyMW): a decrease from 82.9% in neutral conditions to 63.8% in acidic conditions. This larger difference can be due to the positive charges present on both the Dox molecules and the lysine functionalizations under acidic conditions. As for the galactose (1,3-GAMW) and mannose (1,3-MAMW), the difference is smaller but still significant. On the case of galactose, the percentage of adsorbed Dox molecules decreases by around 10%, from 84.7% in neutral conditions to 74.9% in acidic conditions. Images of the simulation that demonstrate this difference can be seen in Fig. 4. Finally, in the case of the mannose functionalized nanotube, the difference is around 7%, decreasing from 84.0% in neutral conditions to 77.3% in acidic conditions. This variance in adsorption levels stems from the protonation of Dox’s amine in acidic conditions. This change makes Dox more hydrophilic and, therefore, more soluble. This change in hydrophilicity, as described in the literature [40, 41], leads to lower adsorption levels of Dox, facilitating its release from the nanotube.

#### Dox adsorption after a change in pH conditions

The previous section demonstrated the differences between adsorption levels at different pH conditions. The next step was to study the unloading of Dox molecules after a switch to acidic conditions. As was described in our previous work [32], this was achieved by protonating the doxorubicin molecules after 300 ns of simulation under neutral conditions and then performing an additional 300 ns of simulation under acidic conditions.

**Fig. 5:**
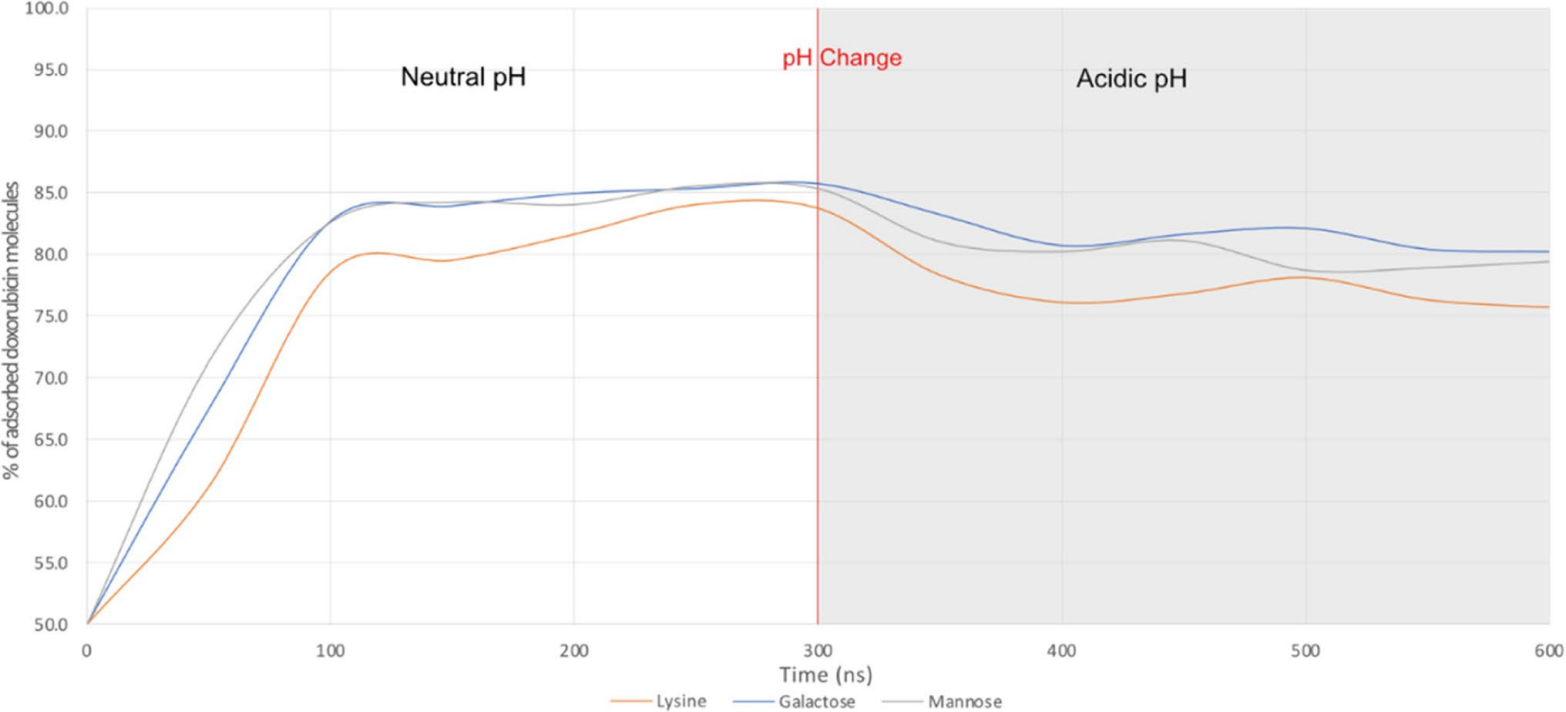
Percentage of Dox molecules adsorbed to each functionalized MWCNT before and after the change in pH conditions

Figure 5 illustrates the percentage of Dox molecules adsorbed onto each functionalized MWCNT over the entire 600 ns simulation period. At 300 ns, following the alteration of pH conditions, there is an immediate decline in the percentage of adsorbed Dox molecules, indicating the release of Dox molecules upon reaching an acidic environment.

**Fig. 6:**
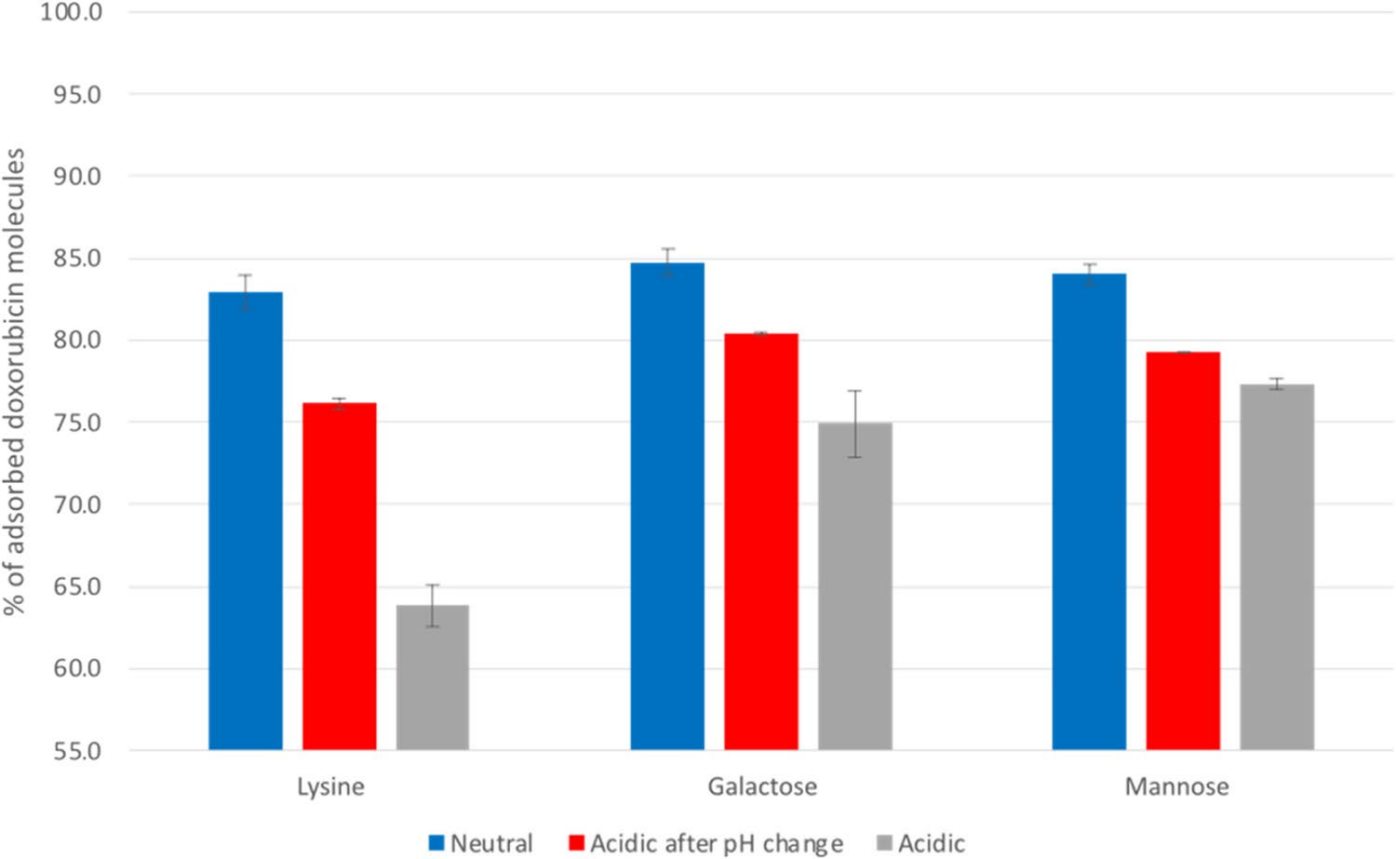
Average percentage of adsorbed Dox molecules in simulations with only neutral conditions, acidic conditions after a change in pH, and only acidic conditions

In Fig. 6, there is a comparison of the adsorption levels in neutral conditions, acidic conditions after a change in pH, and acidic-only conditions. Similar to the comparison between simulations under neutral and acidic conditions, the most significative difference can be seen in the simulations with lysine functionalized MWCNTs. The absorption decreases from 84 to 76%. As for the galactose functionalized MWCNTs, there is a decrease after the change in pH from 86.0 to 80%. Finally, in the simulations with the mannose functionalization, there is a decrease of 85 to 79%. Notably, in all systems, the adsorption values in the simulations with pH change are lower than those in the neutral condition simulations, approximating the values observed in the original simulations conducted under acidic conditions.

The final step was to perform free energy calculations in order to quantify the adsorption capacity of Dox on the different MWCNTs. One Dox molecule per simulation was selected as reference to assess its affinity towards the MWCNTs in the different conditions.

The free energy calculations presented in Table 2 agree with the previous results, demonstrating that, in neutral conditions, there is high affinity of Dox towards all MWCNTs, with ∆*G*_Bind_ values being around −40 kcal/mol. As for the different MWCNTs, it can be seen that sugar-tethered nanotubes have more negative values (−40.0 ± 0.2 and −41.9 ± 0.2 for galactose and mannose vs −39.7 ± 0.1 for lysine) and, therefore, marginally more affinity towards Dox. However, when Dox is protonated, some molecules do not interact with the MWCNT. The same occurrence is observed in simulations after the pH change, with some molecules being released and not interacting with the MWCNT. As expected, when selecting these molecules for the free energy calculations, the results displayed a ∆*G*_Bind_ value close to 0 kcal/mol, reinforcing that some Dox molecules are released from the MWCNTs under acidic conditions.

**Table 2:** Free energy calculations for the interaction with Dox with the functionalized MWCNTs. All values are in kcal/mol

	∆*G*_Bind_ (kcal/mol)	∆*E*_ele_ (kcal/mol)	∆*E*_vdw_ (kcal/mol)	∆*E*_GB_ (kcal/mol)	∆*E*_Surf_ (kcal/mol)
Lysine neutral	− 39.7 ± 0.1	− 4.0 ± 0.3	− 55.4 ± 0.1	20.8 ± 0.2	− 1.0 ± 0.0
Lysine acid after pH change	0.7 ± 0.0	603.5 ± 2.4	− 0.2 ± 0.0	− 602.7 ± 2.4	− 0.0 ± 0.0
Lysine acid	0.0 ± 0.0	455.2 ± 2.6	− 0.0 ± 0.0	− 455.2 ± 2.6	0.0 ± 0.0
Galactose neutral	− 40.0 ± 0.2	− 6.9 ± 0.20	− 58.2 ± 0.2	26.8 ± 0.3	− 1.7 ± 0.0
Galactose acid after pH change	1.2 ± 0.0	45.1 ± 0.1	− 1.7 ± 0.0	− 42.2 ± 0.1	− 0.0 ± 0.0
Galactose acid	− 0.0 ± 0.0	32.9 ± 0.2	− 0.0 ± 0.	− 32.9 ± 0.2	− 0.0 ± 0.0
Mannose neutral	− 41.9 ± 0.2	− 7.00 ± 0.3	− 60.3 ± 0.2	27.1 ± 0.3	− 1.7 ± 0.0
Mannose acid after pH change	0.7 ± 0.0	− 18.2 ± 0.1	− 0.3 ± 0.0	19.9 ± 0.1	− 0.0 ± 0.0
Mannose acid	− 0.0 ± 0.0	− 15.2 ± 0.1	− 0.0 ± 0.0	15.2 ± 0.1	− 0.0 ± 0.0

The method described in this work reveals differences in the adsorption of doxorubicin in functionalized and pristine MWCNTs. The higher adsorption observed with functionalized nanotubes indicates a considerable advantage of using sugar-tethered lysinated functionalizations for the loading of doxorubicin molecules in MWCNTs. Moreover, the explicit water model used in this study can illustrate immediate discrepancies in doxorubicin’s solvation in response to changes in pH. This not only offers further structural insights into the MWCNTs-Dox interaction but also demonstrates the potential capability of these nanotubes for loading doxorubicin under neutral pH conditions and releasing the drug under acidic pH conditions

### Experimental validation

Multiwalled carbon nanotubes were conjugated to lysine via a 1,3-dipolar cycloaddition reaction, which produced lysine-conjugated MWCNTs with a free amino group which were subsequently conjugated to galactose and mannose via the Maillard reaction as shown in the “Computational methods” section. MWCNTs modified with lysine (1,3-LyMW), galactosylated lysine (1,3-GAMW), and mannosylated lysine (1,3-MAMW) were characterized using FT-IR spectroscopy, 1H NMR spectroscopy, X-ray diffractometry, Raman spectroscopy, and scanning electron microscopy. The results demonstrated that sugar-tethered lysinated MWCNTs were successfully prepared (see Supplementary data).

#### Dispersion stability study

**Fig. 7:**
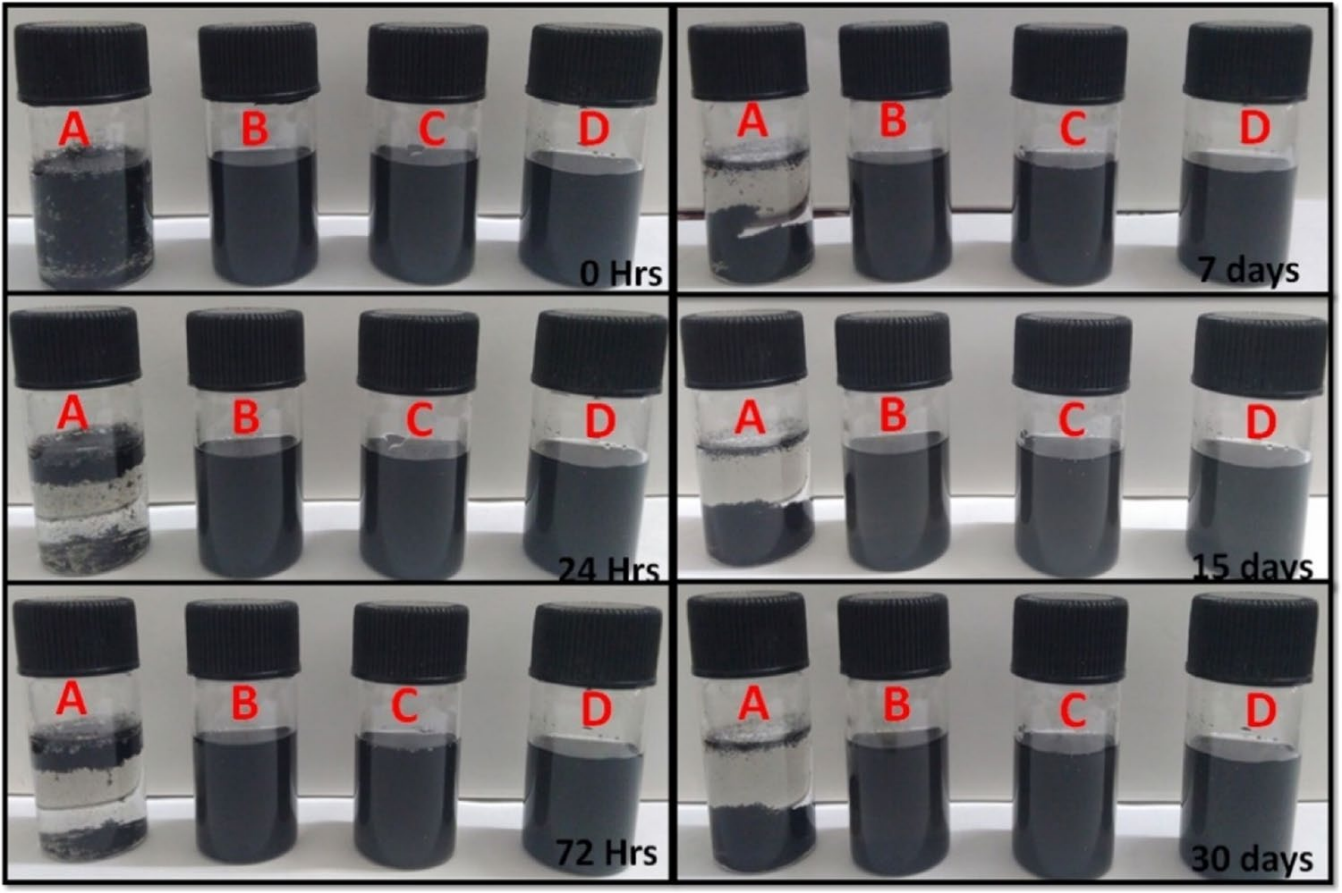
Dispersibility analysis of (**A**) pristine MWCNTs, (**B**) 1,3-lysine-MWCNTs, (**C**) 1,3-galactose-LyMWCNTs, and (**D**) 1,3-mannose-LyMWCNTs

The aqueous dispersibility of MWCNTs modified with lysine and ligands (galactose/mannose) was compared to that of pristine MWCNTs. Ultrasonication was used to disperse PMW, 1,3-LyMW, 1,3-GAMW, and 1,3-MAMW in water for 30 min before monitoring the solution for 30 days to determine the dispersibility of pristine MWCNTs and modified MWCNT formulations, as depicted in Fig. 7. The PMW could not be dispersed well in water after 30 min of sonication, indicating poor dispersibility in aqueous medium due to their high surface energy, which leads them to cluster together and form large bundles [12, 52, 64]. Lysine and galactose/mannose modifications enhanced water dispersibility without causing flocculation. 1,3-LyMW, 1,3-GAMW, and 1,3-MAMW remained stable in water for more than 30 days, demonstrating successful conjugation of lysine and ligands (galactose/mannose) into pristine MWCNTs.

#### Drug loading

**Fig. 8:**
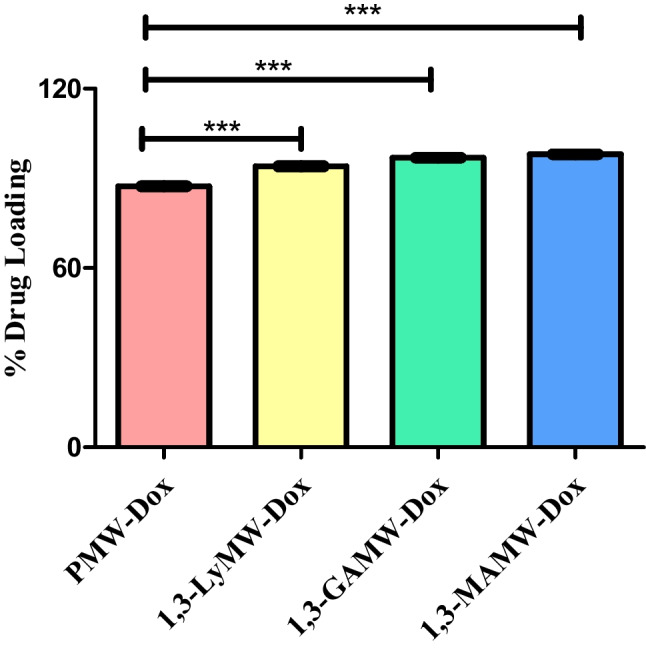
Graphical representation of percentage of Dox loading in pristine MWCNTs, 1,3-lysine-MWCNTs, 1,3-GAMW, and 1,3-MAMW (*n* = 3 and *P* value < 0.001)

The drug loading and release profile are critical aspects in the development and characterization of MWNCTs. Nanoextraction technique was used to determine the Dox loading efficiency of the engineered MWCNTs. Pristine MWCNTs had a Dox loading efficiency of 87.35 ± 2.61%, whereas Dox loaded into lysine-MWCNTs and galactose/mannose MWCNTs exhibited modest improvement, with values of 94.01 ± 0.32, 96.89 ± 1.58, and 98.07 ± 2.34, respectively, for 1,3-LyMW-Dox, 1,3-GAMW-Dox, and 1,3-MAMW-Dox. This is in agreement with the computational predictions, which indicated higher loading capacity for the functionalized MWCNTs. Figure 8 displays a statistically significant *P* value of less than 0.001. As the simulations predicted, Dox may have a higher loading efficiency because of its π-π stacking or hydrophobic interactions with the walls of MWCNTs, both of which are enabled by the presence of aromatic rings in its molecular structure, in addition to performing hydrogen bonds with the functionalized groups of the MWCNTs [54, 55, 65].

#### Drug release mechanism

**Fig. 9:**
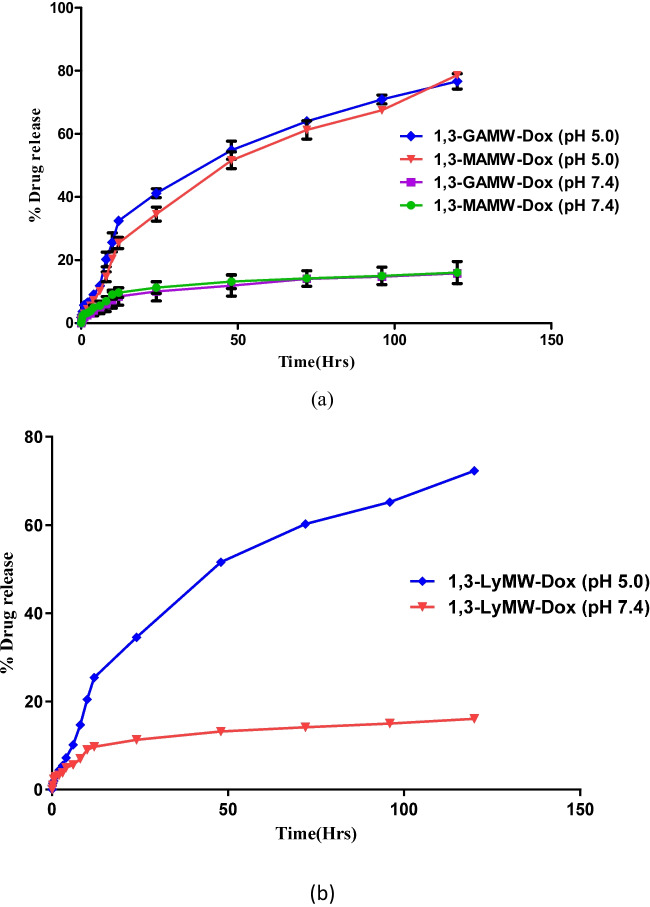
In vitro Dox release profile of Dox-loaded MWCNT formulations at two different pH and result represented at triplicate (*n* = 3)

Dox release from 1,3-LyMW-Dox, 1,3-GAMW-Dox, and 1,3-MAMW-Dox was determined in vitro for 120 h at two different pH values: physiological pH 7.4 and tumor ambient pH 5.0 (Fig. 9). Dox was released at a quicker rate under acidic conditions (pH 5.0) than under physiological conditions (pH 7.4), as was predicted by the MD simulations (Fig. 5). The percentage cumulative Dox release from 1,3-GAMW-Dox and 1,3-MAMW-Dox nanocarriers was reported to be > 75% in acidic pH 5.0 and 20% in physiological pH 7.4, and Dox release from 1,3-LyMW-Dox nanocarriers was found to be 72% in acidic pH 5.0 while 16% in physiological pH 7.4. Dox was released from all nanocarriers more in acidic conditions (pH 5.0) as π-π stacking interaction grew weaker owing protonation of daunosamine group increasing Dox’s pH-dependent hydrophilicity. Since this pH-dependent Dox release behavior is believed to be advantageous for anti-cancer therapy, it is anticipated that the ligand-modified MWCNT formulations, which released Dox only at the tumor site, would be effective against cancer cells but would have no effect on normal cells [53, 56, 66].

#### Cytotoxicity effect of Dox-loaded MWCNTs on *cancer* cells

**Fig. 10:**
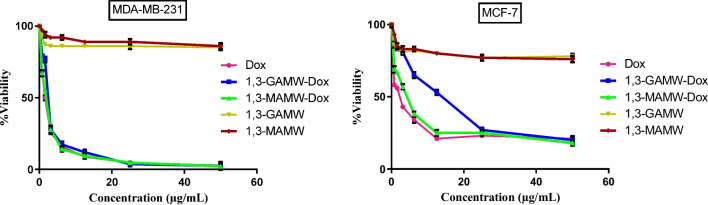
Cytotoxicity effect of Dox, Dox-loaded MWCNTs, and unloaded MWCNT formulations was analyzed by MTT assay in MDA-MB-231 and MCF-7 cell lines. All the data reported as a mean ± SD (*n* = 3). The statistical significances were measured using one-way ANOVA, where “***” represents statistically significant (*P* < 0.0001) and $ represents non-significant (*P* > 0.05) data in comparison to control

MTT assay was used to evaluate the cytotoxic efficacy of 1,3-GAMW-Dox, 1,3-MAMW-Dox, and free Dox on malignant breast cancer cells (MDA-MB-231 and MCF-7). Dox-loaded MWCNT formulations (1,3-GAMW-Dox and 1,3-MAMW-Dox) and free Dox were evaluated in terms of their effects on the viability of MDA-MB-231 and MCF-7 cells, and the results showed that both the treatments reduced cell viability by 50% at concentrations of less than 2.5 µg/ml (Fig. 10 and Table 3). These findings may be explained by the fact that both the formulations (1,3-GAMW-Dox and 1,3-MAMW-Dox) are readily internalized via interaction of galactose/mannose ligands with lectin receptor, resulting in increased Dox accumulation inside the cancer cells and consequently greater cell inhibition. Furthermore, the Dox unloaded formulations 1,3-GAMW and 1,3-MAMW had no significant cytotoxicity, even at higher concentrations (50 µg/ml); the cell viability was found to be nearly 80% and 70% for MDA-MB-231 and MCF-7 cells, respectively. Overall, the prepared formulations 1,3-GAMW-Dox and 1,3-MAMW-Dox induced significant cytotoxicity in breast cancer cells by effectively targeting the lectin receptor which is overexpressed in breast cancer cells.

**Table 3:** Cytotoxicity and cell apoptosis data of Dox and Dox-loaded MWCNT formulations

S. no	Formulations	IC_50_ (µg/ml)		% of apoptosis	
		MDA-MB-231	MCF-7	MDA-MB-231	MCF-7
**1**	Control	–	–	0.5 ± 0.01	0.2 ± 0.03
**2**	Dox	2.0	2.5	21.5 ± 0.06	12.7 ± 0.14
**3**	1,3-GAMW-Dox	1.7	2.3	40.2 ± 0.19	27.4 ± 0.28
**4**	1,3-MAMW-Dox	1.2	1.5	49.7 ± 0.22	44.0 ± 0.30
**5**	1,3-GAMW	–	–	–	–
**6**	1,3-MAMW	–	–	–	–

#### Analysis of Dox and Dox-loaded MWCNT uptake by *cancer* cells

**Fig. 11:**
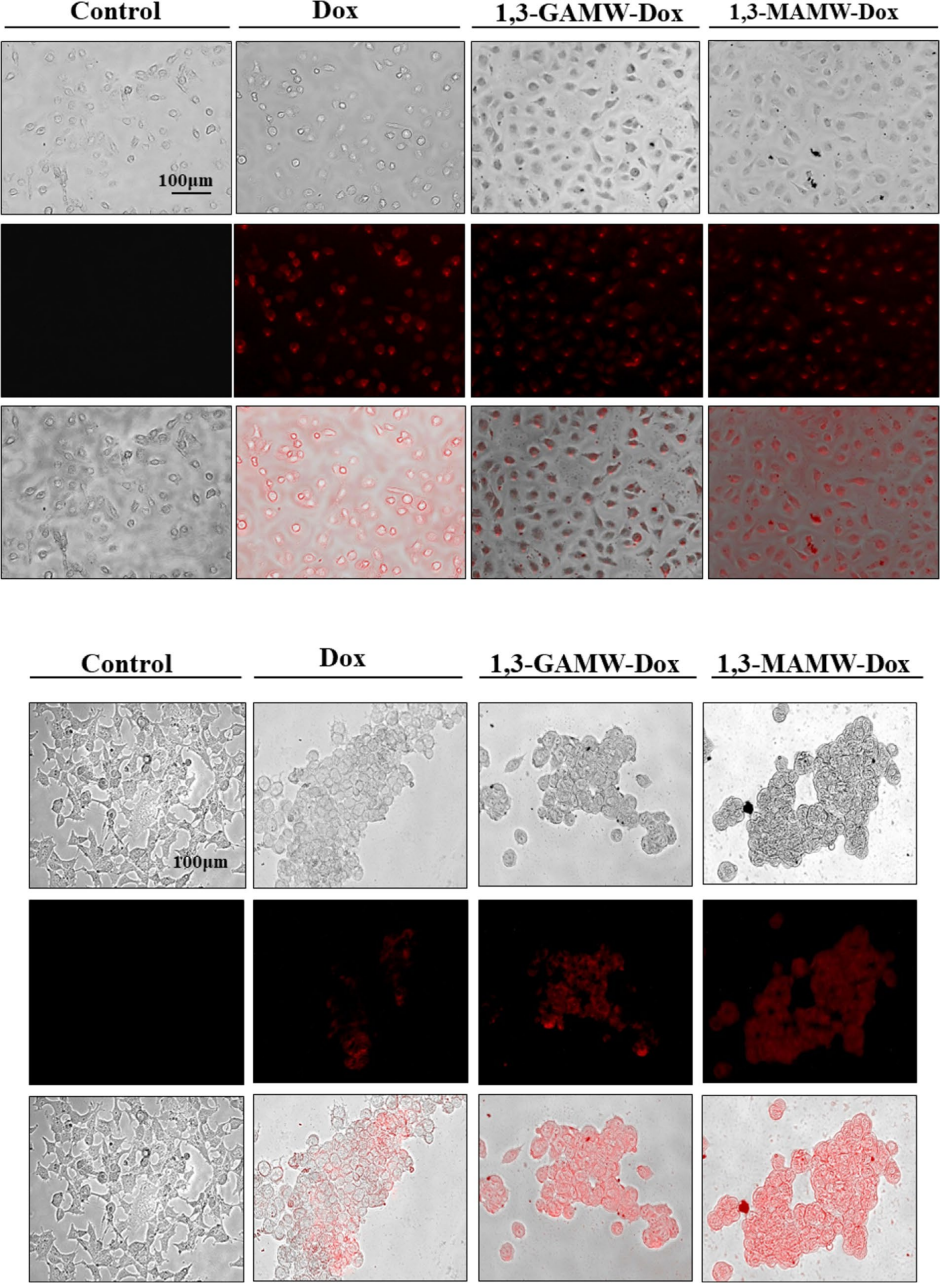
Intracellular uptake of plain Dox and Dox-loaded MWCNT formulations visualized in MDA-MB-231 and MCF-7 cell lines by immunofluorescence

Fluorescence microscopy was used to examine the uptake of plain Dox, 1,3-GAMW-Dox, and 1,3-MAMW-Dox into MDA-MB-231 and MCF-7 breast cancer cells. Uptake of plain Dox and Dox-loaded MWCNTs was assessed by the presence of red fluorescence inside the cells after overnight incubation with the respective MWCNTs (Fig. 11). The red fluorescence intensity of MDA-MB-231 and MCF-7 cells treated with 1,3-GAMW-Dox and 1,3-MAMW-Dox was significantly greater than that of cells treated with Dox alone. These results demonstrate that the galactose/mannose ligands conjugated to 1,3-LyMWCNTs contribute to their internalization into breast cancer cells as previously reported in literature [53, 54].

#### Apoptotic effect of Dox-loaded MWCNTs

**Fig. 12:**
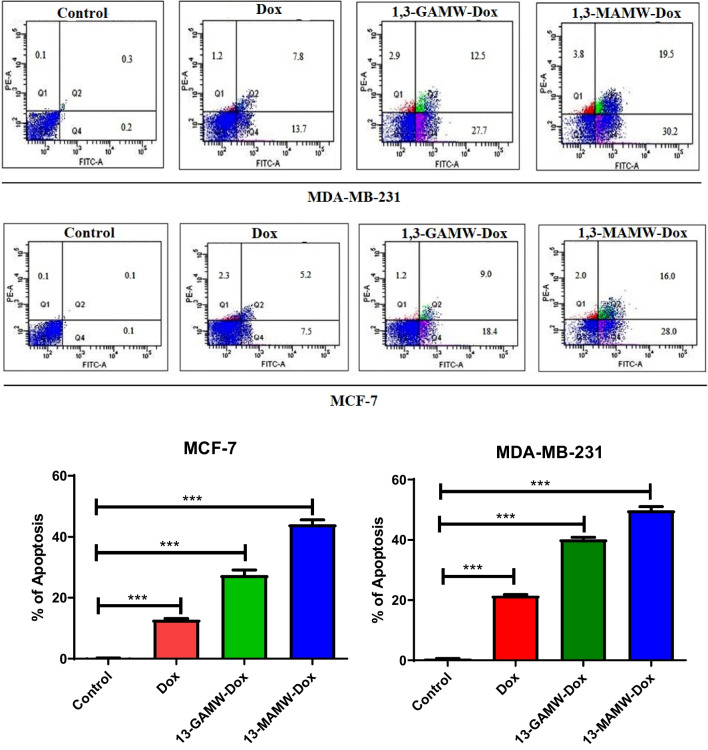
**a** Apoptosis analysis through Annexin-V-FITC/PI dual staining in MDA-MB-231 and MCF-7 cells after being treated with plain Dox and Dox-loaded MWCNT formulations. **b** Bar graph showing the percentage of apoptosis. The results here are represented after performing the experiment in triplicate as mean ± SD, where *** represents statistically significant (*P* < 0.0001)

The rate of apoptotic potentiality of Dox-loaded MWCNTs in comparison to free Dox in MDA-MB-231 and MCF-7 breast cancer cells was determined by FACS (Fig. 12). Our data demonstrate that Dox-loaded MWCNT formulations induce a greater percentage of cell apoptosis in both cell types as compared to free Dox. In MDA-MB-231, the percentage of apoptosis was found to be 21.5 ± 0.06, 40.2± 0.19, and 49.7 ± 0.22 for free Dox, 1,3-GAMW-Dox, and 1,3-MAMW-Dox, respectively, while in MCF-7, the corresponding values were 12.7 ± 0.03, 27.4 ± 0.14, and 44.0 ± 0.28, respectively. The observed results are statistically significant, as the *P* value is less than 0.001.

### Comparison of in silico and in vitro results

Drug loading studies performed *in silico* and *in vitro* demonstrated that functionalization of MWCNTs with lysine and ligands leads to an increased Dox loading capacity when compared to pristine MWCNTs. This is because functionalized MWCNTs interact with Dox via π-π stacking and hydrogen bonding interactions, whereas pristine MWCNTs only interact through π-π stacking. MD studies predicted higher adsorption under neutral conditions and a release of Dox molecules under acidic conditions. The drug release studies corroborated the computational predictions and demonstrated that Dox was released at a higher percentage at intratumoral pH 5.0 compared to physiological pH 7.4. Dox was released from both ligand-conjugated MWCNTs under more acidic conditions due to the protonation of the daunosamine group, which reduced the π-π stacking interaction between the surface of the MWCNT and the polycyclic ring of the drug and increased the pH-dependent hydrophilicity of Dox. *In silico* studies aided in understanding the interactions that contribute to higher drug loading as well as the mechanism underlying higher Dox release at intratumoral pH, which in vitro experiments were unable to discern. The ability to predict experimental outcomes while enhancing comprehension of the interactions between the drug and the nanotubes underscores the effectiveness of this computational approach. The high drug loading and intratumoral pH-sensitive Dox release behavior is believed to be advantageous for anti-cancer therapy as tumor-targeted delivery of doxorubicin can be achieved by using low quantity of functionalized MWCNTs.

## Conclusion

In this work, MD simulations were used to study novel and highly efficient sugar-tethered MWCNT nanocarriers which use lysine as an inexpensive and biocompatible linker. The computational method, which calculates the number of water molecules surrounding each Dox molecule in order to estimate its solubility and adsorption to the nanotubes, predicted that functionalized MWCNTs had a higher percentage of Dox loading than pristine MWCNTs. This is due to improved interaction between Dox and the surface of functionalized MWCNTs as in addition to π-π stacking, hydrogen bonds are also possible. In addition, MD simulations shed light on the intratumoral pH-specific release of Dox from functionalized MWCNTs, which is induced by protonation of the daunosamine moiety. The computational results were validated by the in vitro studies, where the modified functionalized MWCNTs showed good aqueous dispersibility and enhanced drug loading capacity compared to the pristine MWCNTs. In vitro release studies indicated that the Dox-loaded functionalized MWCNTs exhibited pH-sensitive release of Dox at intratumoral pH 5.0 with minimal release at physiological pH 7.4, as predicted computationally. To more completely access the potential of these structures, the in vitro cytotoxic ability was analyzed using MTT assay and apoptosis by Annexin-V-FITC/PI flow cytometry on breast cancer cells indicated the superior anti-cancer efficacy of Dox-loaded functionalized MWCNTs. Finally, cellular uptake studies with Dox-loaded functionalized MWCNT demonstrated enhanced cellular uptake indicative of effective ligand-mediated lectin receptor targeting. The MD simulations performed in this work, together with the experimental validation, suggest that sugar-tethered lysine functionalized multiwalled carbon nanotubes offer a viable low-cost, non-toxic nanoplatform for targeted delivery of anti-cancer drugs to breast cancer.

### Supplementary Information

Below is the link to the electronic supplementary material.

Supplementary file1 (PDF 2316 KB)

## Data Availability

The datasets generated during and/or analysed during the current study are available from the corresponding author on reasonable request.
