# Supplementary material for: In silico-guided discovery and in vitro validation of novel sugar-tethered lysinated carbon nanotubes for targeted drug delivery of doxorubicin
Source: J Mol Model. 2024 Jul 10;30(8):261. doi: 10.1007/s00894-024-06061-5 (PMC11236919; doi:10.1007/s00894-024-06061-5)
Supplement: Supplementary file 1 — Supplementary file1 (PDF 2316 KB) [file 894_2024_6061_MOESM1_ESM.pdf]

In Silico-Guided Discovery and In Vitro Validation of Novel Sugar-Tethered Lysinated Carbon Nanotubes for Targeted Drug Delivery of Doxorubicin

Chanchal Kiran Thakur[a], Fábio G. Martins[b], Chandrabose Karthikeyan[a], Subhasmita Bhal[c], Chanakya Nath Kundu[c], , N.S. Hari Narayana Moorthy[a]*, Sérgio F. Sousa[b]*

[a]*Cancept Therapeutics Laboratory, Department of Pharmacy, Indira Gandhi National Tribal University, Lalpur, Amarkantak (MP)-484887, India.*

[b]*LAQV/REQUIMTE, BioSIM-Departamento de Biomedicina, Faculdade de Medicina, Universidade do Porto, 4200-319 Porto, Portugal;*

[c]*Cancer Biology Division, School of Biotechnology, KIIT deemed to be University, Campus-11, Patia, Bhubaneswar, Odisha, 751024, India.*

Chanchal Kiran Thakur: chanchalthakur2516@gmail.com ORCID: 0000-0001-9109-6498

Fábio G. Martins: fabiogfmartins@gmail.com ORCID: 0000-0001-6257-8885

Chandrabose Karthikeyan: karthikeyanchandrabose@gmail.com ORCID: 0000-0002-9074-9554

Subhasmita Bhal: bhalsubhasmita@gmail.com ORCID: 0000-0002-4394-0399

Chanakya Nath Kundu: cnkundu@kiitbiotech.ac.in ORCID: 0000-0003-0297-1030

N.S. Hari Narayana Moorthy: hari.nmoorthy@gmail.com ORCID: 0000-0003-2300-4074

Sérgio F. Sousa: sergiofsousa@med.up.pt ORCID: 0000-0002-6560-5284

*For Correspondence hari.nmoorthy@gmail.com, sergiofsousa@med.up.pt

Supplementary Information

Methods

Conjugation of Boc-Lysine with pristine MWCNTs by 1,3-dipolar cycloaddition reaction:

Two hundred milligram of pristine MWCNTs was dispersed in 200 ml of dimethylformamide and added to this was added Boc-Lysine and paraformaldehyde (1:1 molar ratio) every 24 hr upto 5 days. The reaction suspension was stirred for 120 hr at 130ºC. Upon completion, the unreacted MWCNTs which remained insoluble in dimethylformamide was filtered out using Millipore polytetrafluoroethylene filter (0.22 µm pore size) and washed with deionized water. The resulting brown filtrate was evaporated and concentrated under reduced pressure to give a dark brown oily liquid which was collected and then dialyzed (dialysis membrane: 12 kDa molecular weight cutoff, HiMedia Laboratories, India) in deionized water for 24 hr. The retentate was collected and acidified (pH=4) with hydrochloric acid, then washed once with ethyl acetate and chloroform. Finally, the aqueous layer was collected and basified with sodium hydroxide at 50ºC till it becomes cloudy. The cloudy solution was then allowed to cool in order to form a layer of brown powder. The powder layer was separated by filtration and washed with water until it became neutral, after which the solid materials were collected and dried under vacuum. The obtained Boc-lysine functionalized MWCNTs were stirred for 2 hr at 50ºC in trifluoroacetic acid to remove the BOC group. The lysine functionalized MWCNTs recovered after evaporating the solvent was washed with deionized water and dried using vacuum desiccator [1].

Covalent functionalization of sugar moieties with 1,3-Lysine-MWCNTs:

1,3-LyMW (100 mg) were dispersed into sodium acetate buffer (pH=4) then sonicated for 20 min. Sugar ligands (galactose and mannose) 275 mg was added into 1,3-LyMW suspension and the reaction mixture was then sonicated for few minutes, followed by continuous stirring for 3 days at room temperature. Upon completion of 3 days, the suspension was centrifuged at 4°C for 30 min at 10,000 and the supernatant was decanted. The residue was dialyzed overnight in deionized H_2O using a dialysis bag to remove unbound or free ligands, then centrifuged for 15 min at 10,000 rpm, 4°C to remove the supernatant and collect the final products (1,3-galactose-MWCNTs/ 1,3-mannose-MWCNTs) [2–4].

Fourier Transform Infrared Spectroscopy:

The pristine MWCNTs (PMW) and modified MWCNTs (1,3-LyMW, 1,3-GAMW & 1,3-MAMW) were characterized by FT-IR spectroscopy (M/S Bruker, vertex 70, Optic GmbH, Germany) and FT-IR spectra were recorded in the range 4000-600 cm^{-1} using KBr pellet method.

Nuclear Magnetic Resonance Spectroscopy (^{1}H NMR):

Lysine-functionalized MWCNTs (1,3-LyMW) and sugar-tethered lysine-functionalized MWCNTs (1,3-GAMW & 1,3-MAMW) were dispersed in d6-DMSO, and ^{1}H NMR spectra were acquired using an NMR spectrometer (Avance NEO 500MHz FT-NMR spectrometer, M/S Bruker, Switzerland).

X-Ray Diffraction Study:

The XRD analysis of 1,3-LyMW, 1,3-GAMW & 1,3-MAMW was characterized by powder-X-Ray diffraction (M/S Malvern PANalytical, X Pert^3 Powder, ALMELO, Netherlands) and the diffraction values were measured at 2Θ values between the range of 10-50.

Raman Spectroscopy:

The Raman spectra (Horiba Joib Yuon-CCD detector, France) analysis of pristine MWCNTs, 1,3-LyMW, 1,3-GAMW & 1,3-MAMW was done at Central Research Facility, IIT Kharagpur, West Bengal, India. In the range of 1000–2000 cm^{-1} and the overall spectral resolution of the set-up was 1cm^{-1}.

Field Emission Scanning Electron Microscope (FE-SEM):

The FE-SEM (Supra 55, Carl Zeiss with InCA X-ACT EDS Oxford Instruments) surface morphology analysis of pristine MWCNTs and modified MWCNTs was done at the UGC-DAE Consortium for Scientific Research, Kalpakkam, Chennai, India.

Particle size distribution and Zeta potential studies:

The particle size, polydispersity index, and Zeta potential of modified MWCNTs were analysed using particle size analyzer (Litesizer 500 from Anton PAAR, Graz, Austrian, USA). The samples were analysed in triplicates and the mean values are reported.

Results

FT-IR Spectroscopy

Figure S1 shows the FTIR spectra of both pristine MWCNTs and MWCNTs modified with lysine (1,3-LyMW), galactosylated lysine (1,3-GAMW) and mannosylated lysine (1,3-MAMW). The lysine modifications resulted in a distinct peak spectrum compared to the unaltered MWCNTs. The FT-IR spectra of 1,3-Lysine-MWCNTs exhibit a peak at 3445 cm^{-1} for N-H stretching, 1538 cm^{-1} and 1413 cm^{-1} for N-H bending, and 1146 cm^{-1} for C-N stretching, confirming the presence of an amino group [4, 5]. Similarly for 1,3-GAMW & 1,3-MAMW, the broad peaks at 3332 cm^{-1} and 3335 cm^{-1} correspond to the O-H stretching of the hydroxyl group of galactose and mannose, whereas the sharp peaks at 2970 cm^{-1} and 2974 cm^{-1} indicate N-H stretching, 1604 cm^{-1} and 1602 cm^{-1} indicate N-H bending, and 1454 cm^{-1} and 1452 cm^{-1} indicate -C-N- bending. All of these peaks suggest the successful galactosylation and mannosylation of 1,3-Lysine-MWCNTs *via* Schiff's reaction [2, 6].

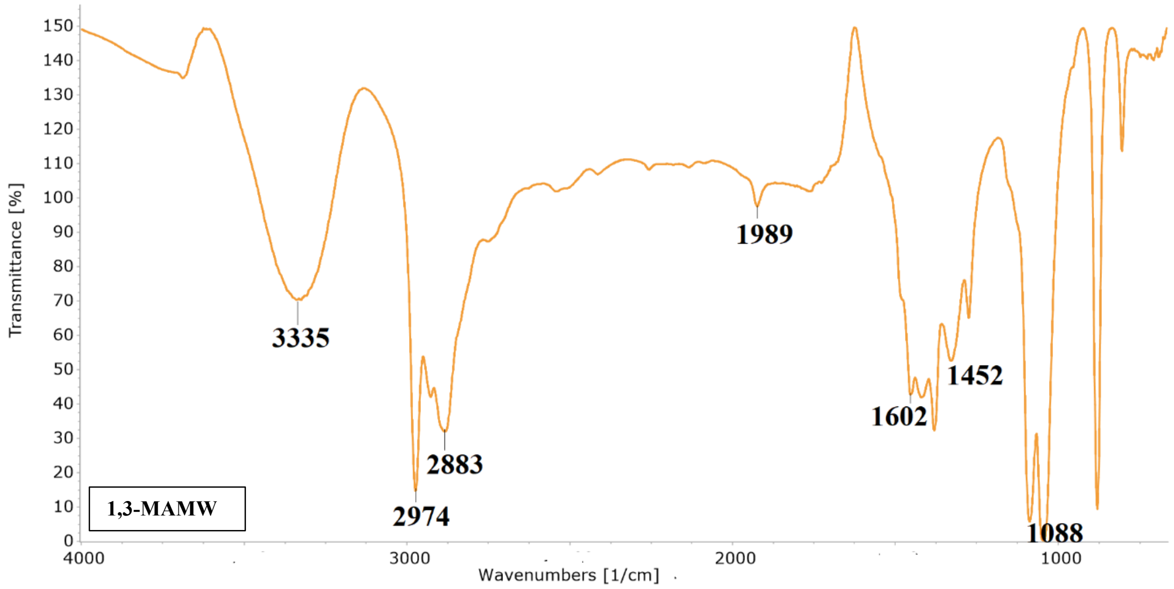

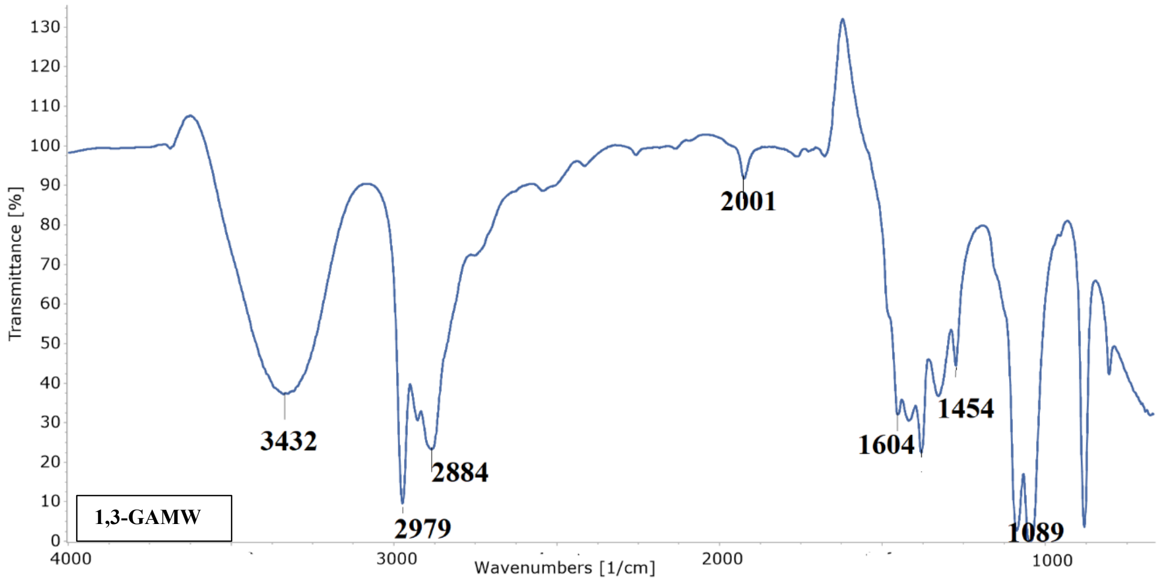
1,3-GAMW
Transmittance [%]
Wavenumbers [1/cm]
3432
2979
2884
2001
1604
1454
1089

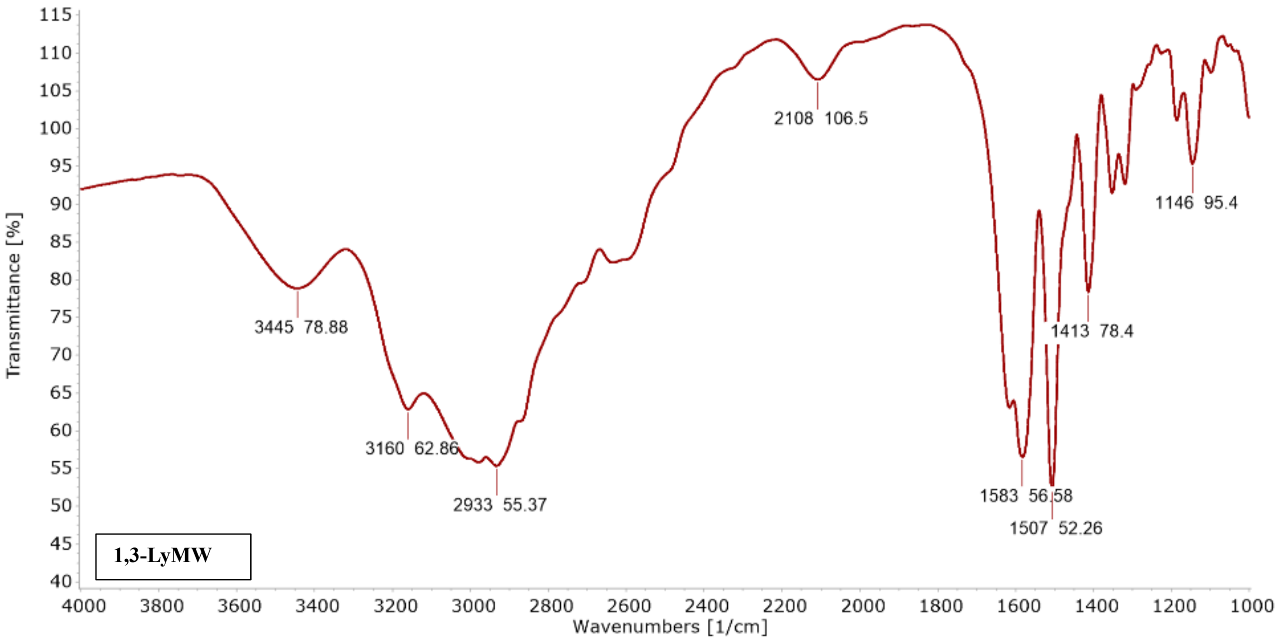
1,3-LyMW
Transmittance [%]
Wavenumbers [1/cm]
3445 78.88
3160 62.86
2933 55.37
2108 106.5
1583 56.58
1507 52.26
1413 78.4
1146 95.4

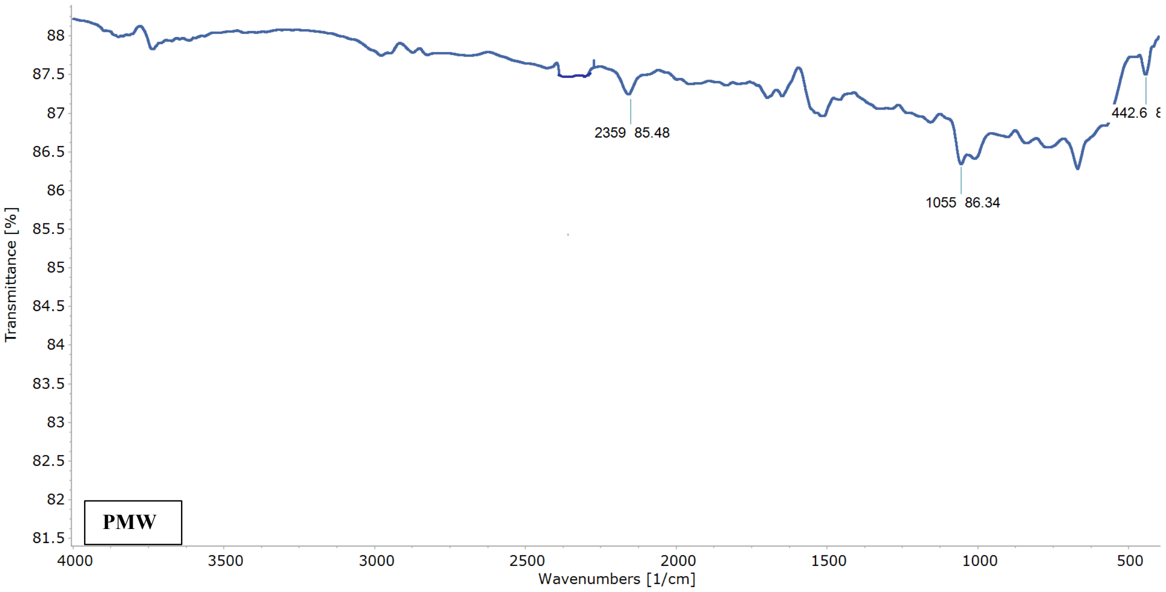

Fig. S1 *FT-IR graphs of pristine MWCNTs, 1,3-Lysine-MWCNTs, 1,3-Galactose-LyMWCNTs and 1,3-Mannose-LyMWCNTs*

^{1}H NMR Spectroscopy

Results from 1H NMR spectra (Figure S2) confirmed the lysine functionalization of MWCNTs and the efficient galactose/mannose conjugation to lysine functionalized MWCNTs. The broad signals at 1.15-2.4 ppm can be attributed to the methylene proton of lysine and 7.11 ppm showed as 'NH_2' proton of lysine respectively, demonstrating that lysine has been effectively conjugated in MWCNTs, almost similar results was observed as the previous reported articles. Galactose/mannose conjugated lysine functionalized MWCNTs showed a singlet peak intensity between 8.49 and 8.50 ppm, indicating the formation of an imine bond between the aldehyde group of galactose/mannose and the ε-amino group of lysine conjugated MWCNTs. A peak between 1.0 and 3.0 ppm, corresponding to the methylene and -OH protons of galactose/mannose, demonstrated efficient conjugation of these sugars into lysine-MWCNTs [7, 8].

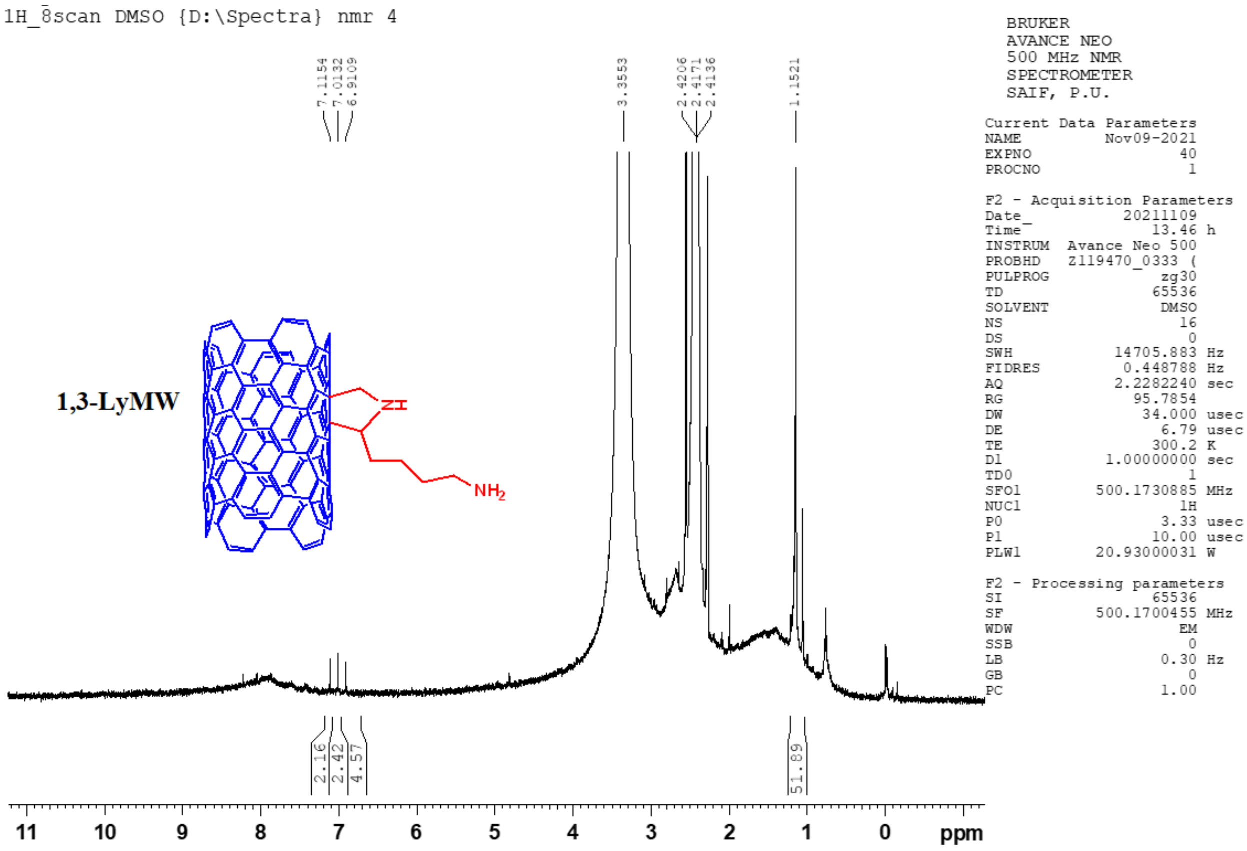

1H_8scan DMSO {D:\Spectra} nmr 4
7.1154
7.0132
6.9109
3.3553
2.4206
2.4171
2.4136
1.1521
1,3-LyMW
NH
NH_2
2.16
2.42
4.57
51.89
11 10 9 8 7 6 5 4 3 2 1 0 ppm
BRUKER
AVANCE NEO
500 MHz NMR
SPECTROMETER
SAIF, P.U.
Current Data Parameters
NAME Nov09-2021
EXPNO 40
PROCNO 1
F2 - Acquisition Parameters
Date_ 20211109
Time 13.46 h
INSTRUM Avance Neo 500
PROBHD Z119470_0333 (
PULPROG zg30
TD 65536
SOLVENT DMSO
NS 16
DS 0
SWH 14705.883 Hz
FIDRES 0.448788 Hz
AQ 2.2282240 sec
RG 95.7854
DW 34.000 usec
DE 6.79 usec
TE 300.2 K
D1 1.00000000 sec
TD0 1
SFO1 500.1730885 MHz
NUC1 1H
P0 3.33 usec
P1 10.00 usec
PLW1 20.93000031 W
F2 - Processing parameters
SI 65536
SF 500.1700455 MHz
WDW EM
SSB 0
LB 0.30 Hz
GB 0
PC 1.00

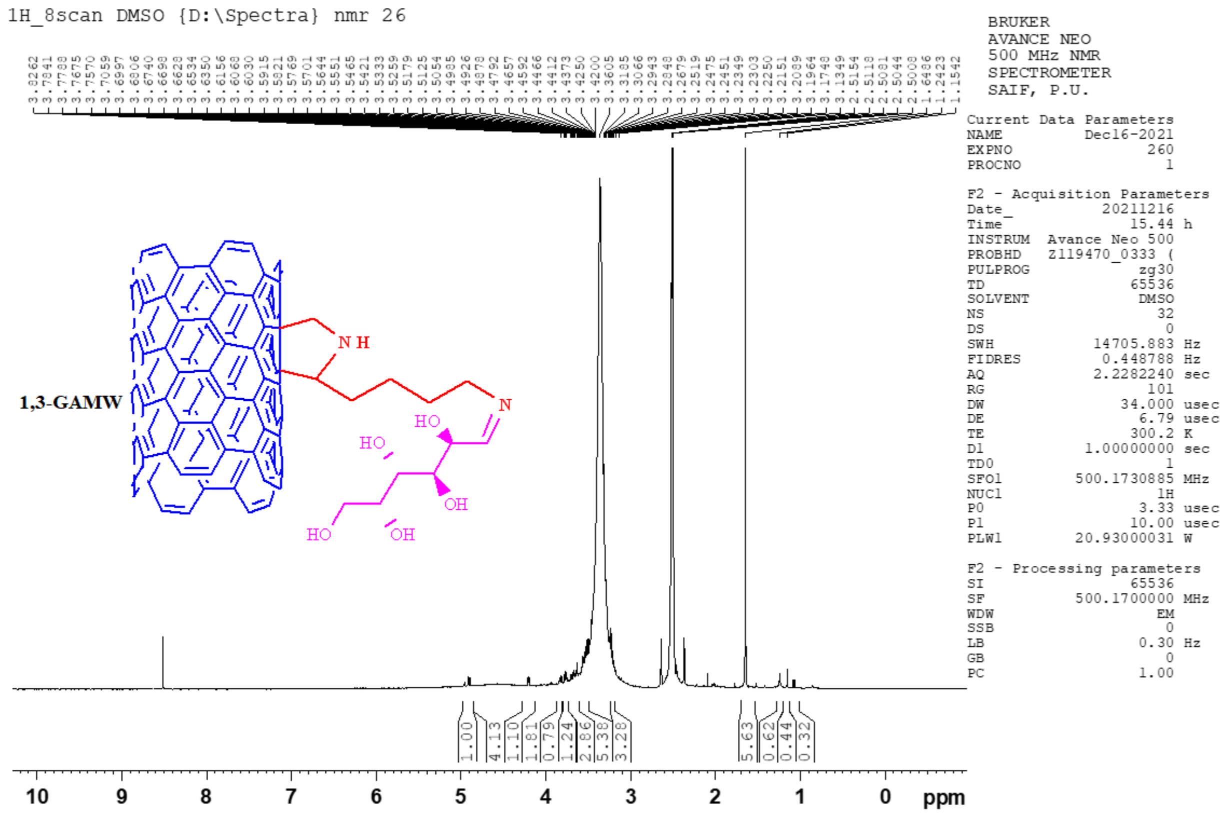

1H_8scan DMSO {D:\Spectra} nmr 26
1,3-GAMW
N H
N
HO
HO
OH
HO
OH
1.00
4.13
1.10
1.81
0.79
1.24
2.86
5.38
3.28
5.63
0.62
0.44
0.32
10 9 8 7 6 5 4 3 2 1 0 ppm
BRUKER
AVANCE NEO
500 MHz NMR
SPECTROMETER
SAIF, P.U.
Current Data Parameters
NAME Dec16-2021
EXPNO 260
PROCNO 1
F2 - Acquisition Parameters
Date_ 20211216
Time 15.44 h
INSTRUM Avance Neo 500
PROBHD Z119470_0333 (
PULPROG zg30
TD 65536
SOLVENT DMSO
NS 32
DS 0
SWH 14705.883 Hz
FIDRES 0.448788 Hz
AQ 2.2282240 sec
RG 101
DW 34.000 usec
DE 6.79 usec
TE 300.2 K
D1 1.00000000 sec
TD0 1
SFO1 500.1730885 MHz
NUC1 1H
P0 3.33 usec
P1 10.00 usec
PLW1 20.93000031 W
F2 - Processing parameters
SI 65536
SF 500.1700000 MHz
WDW EM
SSB 0
LB 0.30 Hz
GB 0
PC 1.00

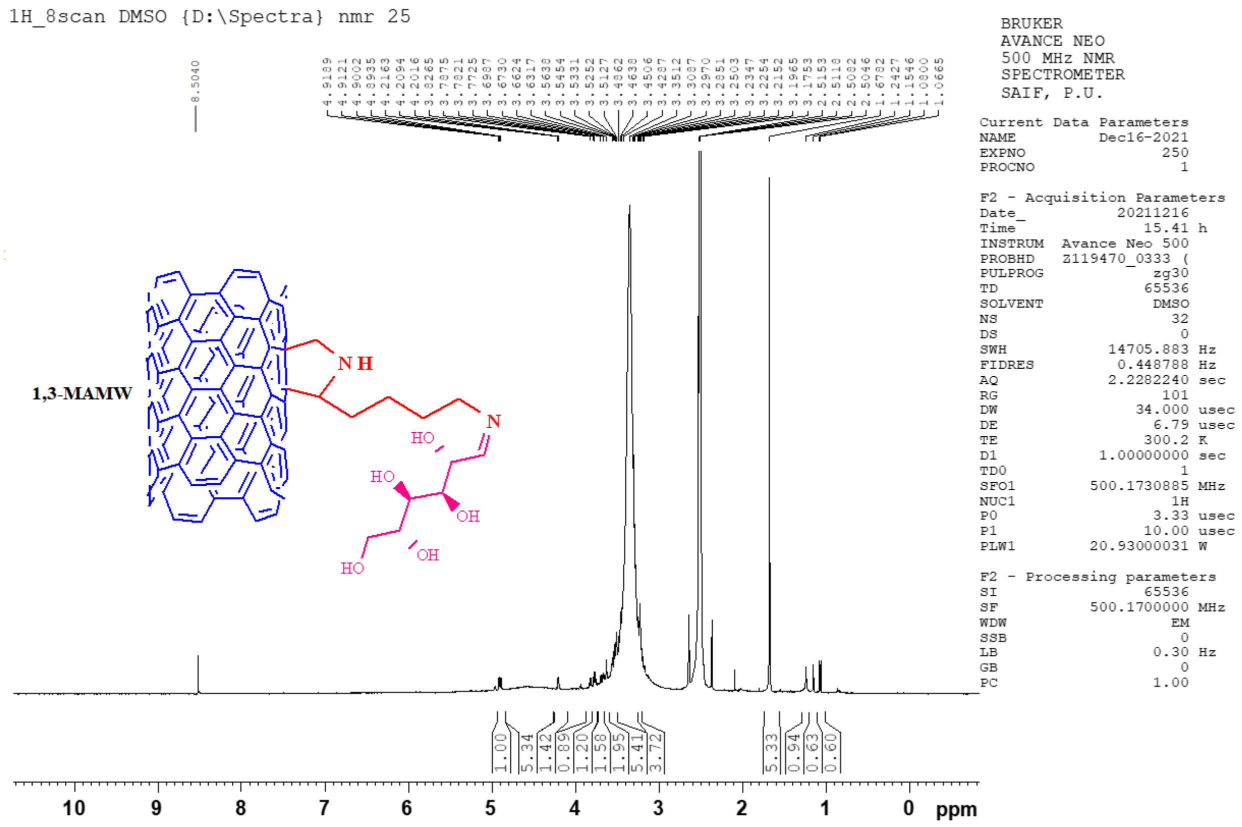

Fig. S2 *H1NMR spectra of 1,3-Lysine-MWCNTs, 1,3-Galactose-LyMWCNTs and 1,3-Mannose-LyMWCNTs*

X-Ray Diffraction Study

The XRD graph of lysine-conjugated MWCNTs in Figure S3 displays a peak at 25.58. In addition, the galactose/mannose-modified lysine-MWCNTs XRD pattern revealed several diffraction peaks at 29.62, 25.54, 22.46, 18.94, 16.82, and 11.38 (1,3-GAMW) and 29.60, 25.14, 22.46, 18.98, 16.84, and 11.38 (1,3-GAMW) (1,3-MAMW). These peaks corresponded to the successful conjugation of galactose/mannose with lysine-MWCNTs [9–12].

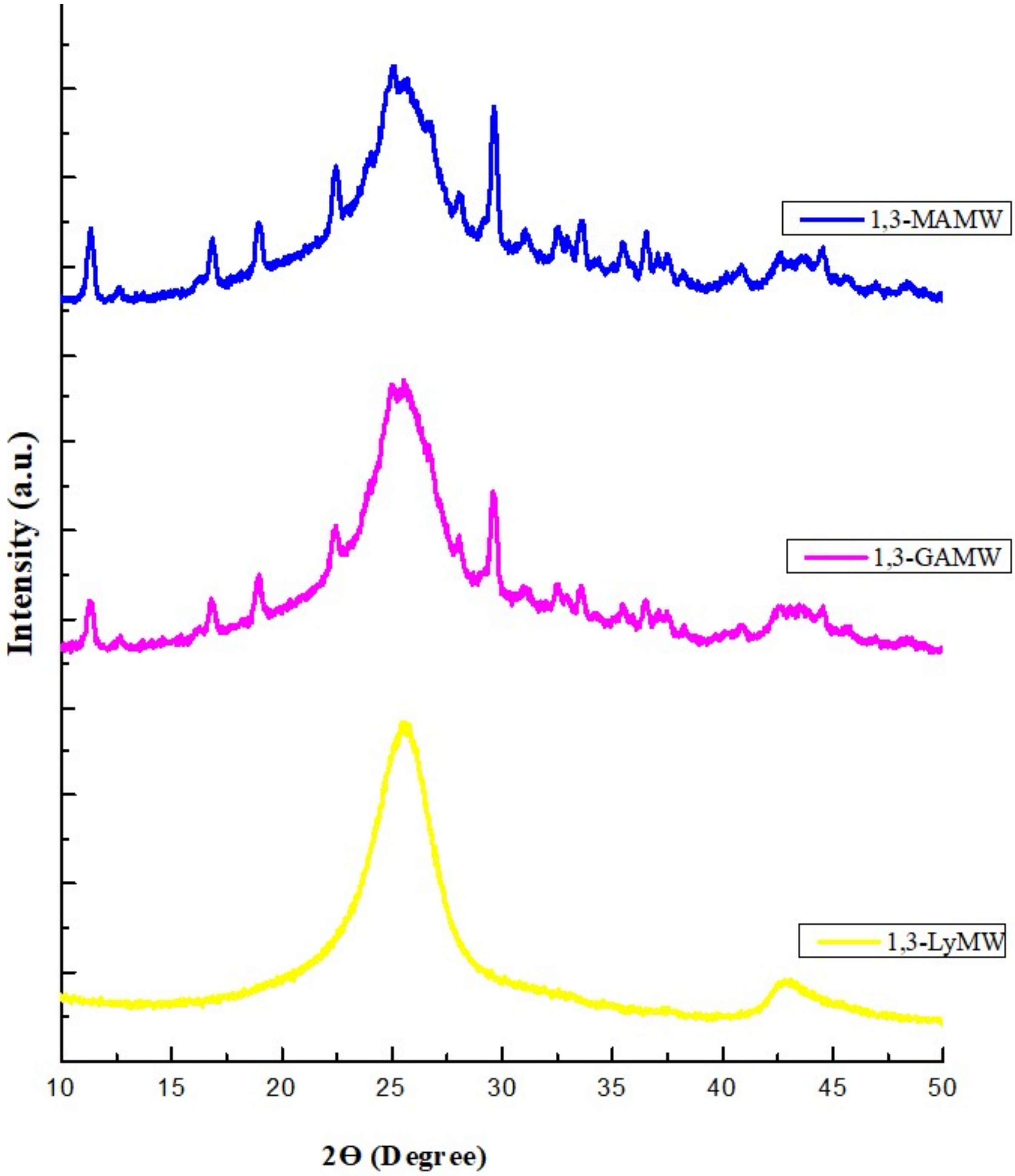

Fig. S3 *XRD pattern of 1,3-Lysine-MWCNTs, 1,3-Galactose-LyMWCNTs and 1,3-Mannose-LyMWCNTs*

Raman Spectroscopy

Raman spectroscopy is typically employed for identifying structural changes in pharmaceutical formulations [13, 14]. Figure S4 shows the Raman spectra of both pristine MWCNTs and modified MWCNTs. The Raman spectra of pristine and functionalized MWCNTs exhibited two distinctive bands: D-band & G-band at 1334 cm^{-1} and 1566 $cm^{-1,}$ respectively. D-band corresponds to sp^2 hybridized atoms, whereas the G-band corresponds to the structural integrity of the sp^2 hybridized atoms in the MWCNTs. The I_D/I_G ratio is determined based on the D and G-bands, which correlate to the sp^2-hybridized or structural integrity of the sp^2-hybridized carbon atoms in

nanoformulations [15]. The I_D/I_G ratios for PMW, 1,3-LyMW, 1,3-GAMW, and 1,3-MAMW were determined to be 1.01, 1.20, 0.83, and 0.81, respectively. Lysine-functionalized MWCNTs have a greater I_D/I_G ratio than pristine MWCNTs, indicating a strong sidewall conjugation of molecules. However, the I_D/I_G ratio of 1,3-GAMW and 1,3-MAMW was lower than that of PMW and 1,3-LyMW, suggesting an extension of covalent conjugation [2]

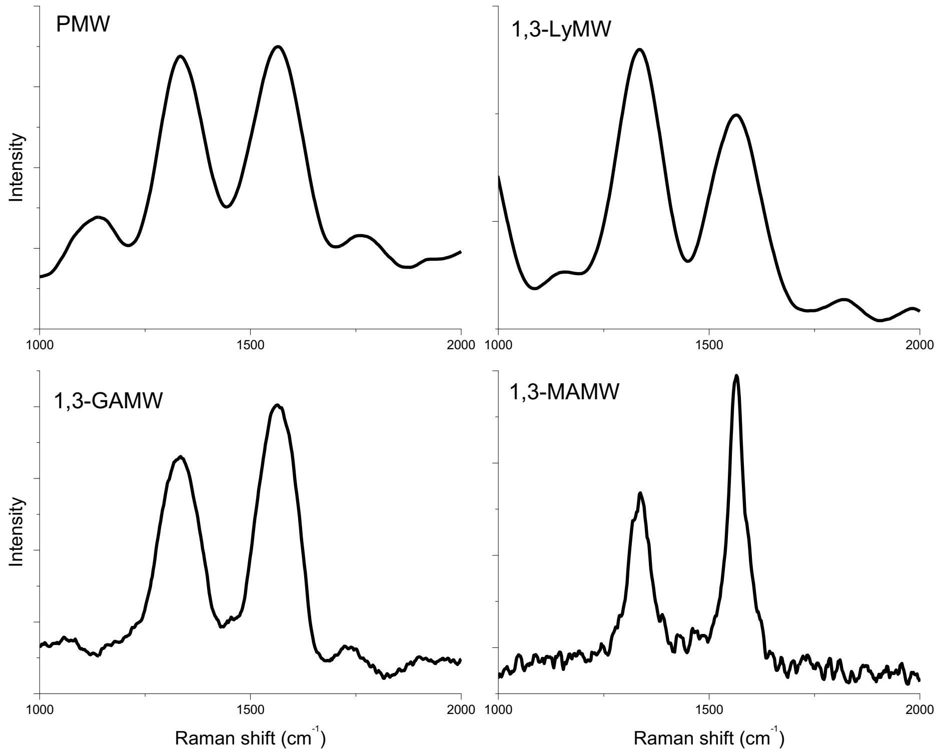

Fig, S4 *Raman-spectra of the pristine MWCNTs and functionalized MWCNTs*

FE-SEM Morphology

FE-SEM was used to evaluate the surface morphology of both pristine MWCNTs and modified MWCNTs; Figure S5 shows the surface morphology of PMW, 1,3-LyMW, 1,3-GAMW, and 1,3-MAMW. Surface morphologies of pristine MWCNTs and functionalized MWCNTs were remarkably similar, indicating that PMW formed during the functionalization procedure with lysine and galactose/mannose had little effect on the structures of either kind of MWCNTs, consistent with prior findings [16–18]. Furthermore, FE-SEM images of functionalized MWCNTs with lysine or galactose/mannose revealed a rough surface, in contrast to the relatively smooth surface of the pristine MWCNTs. The PMW, 1,3-LyMW, 1,3-GAMW, and 1,3-MAMW were agglomerated because as water evaporates during the drying process, the distance between the nanotubes expands, causing them to interact through van der Waal's forces [4].

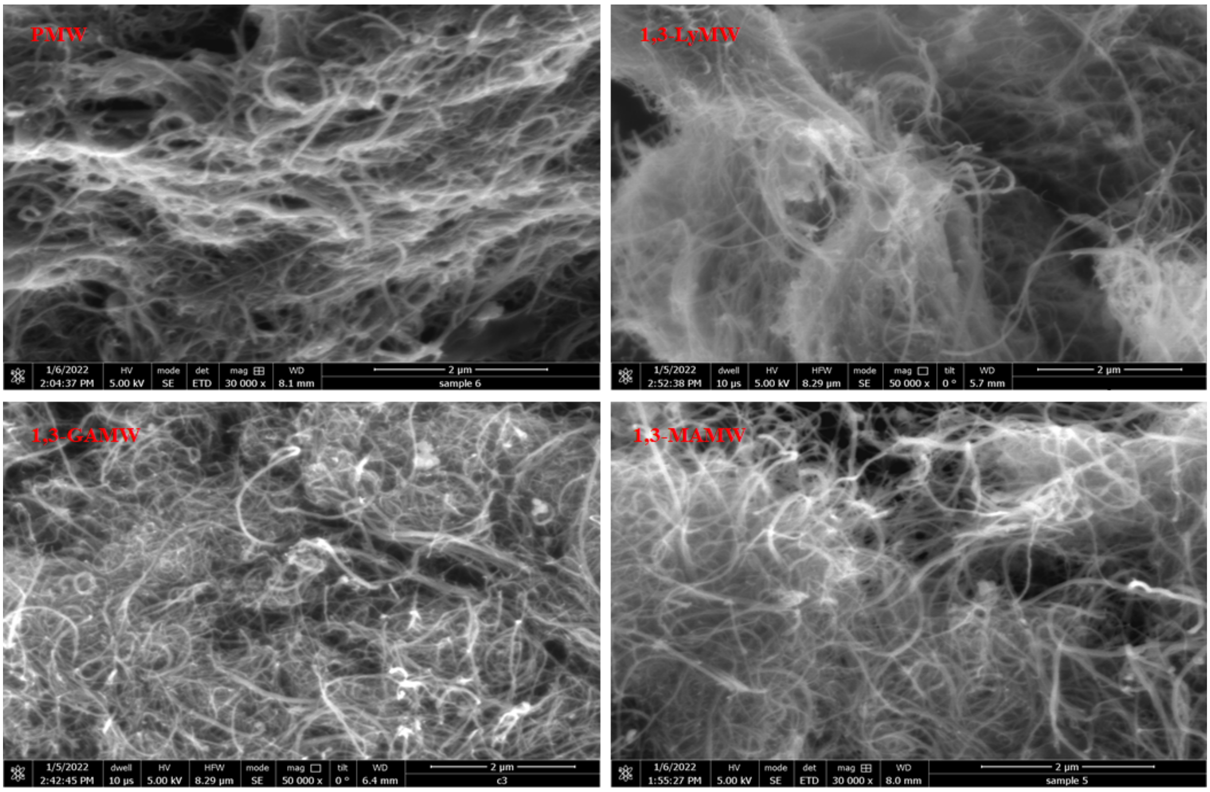

Fig. S5 *FE-SEM images of pristine MWCNTs and modified MWCNTs*

Particle size distribution

The particle size (PS) and polydispersity index (PDI) of pristine MWCNTs and modified MWCNTs were determined, and the findings are shown in Figure S6. The particle sizes of pristine MWCNTs, 1,3-Lysine-MWCNTs, Dox-loaded 1,3-Galactose MWCNTs, and Dox-loaded 1,3-Mannose MWCNTs were determined to be 115.84±1.35 nm, 142.63±0.76 nm, 181.02±1.87 nm, and 189.78±2.75 nm, respectively. The PDI measurements were ranging from 0.15 to 0.30. These findings suggest that the increase in size could be interpreted as a sign of the intended attachment of lysine, galactose/mannose, and adsorption of Dox in MWCNTs. The measured PDI values were less than 0.3, indicating that the system was fairly reliable [19].

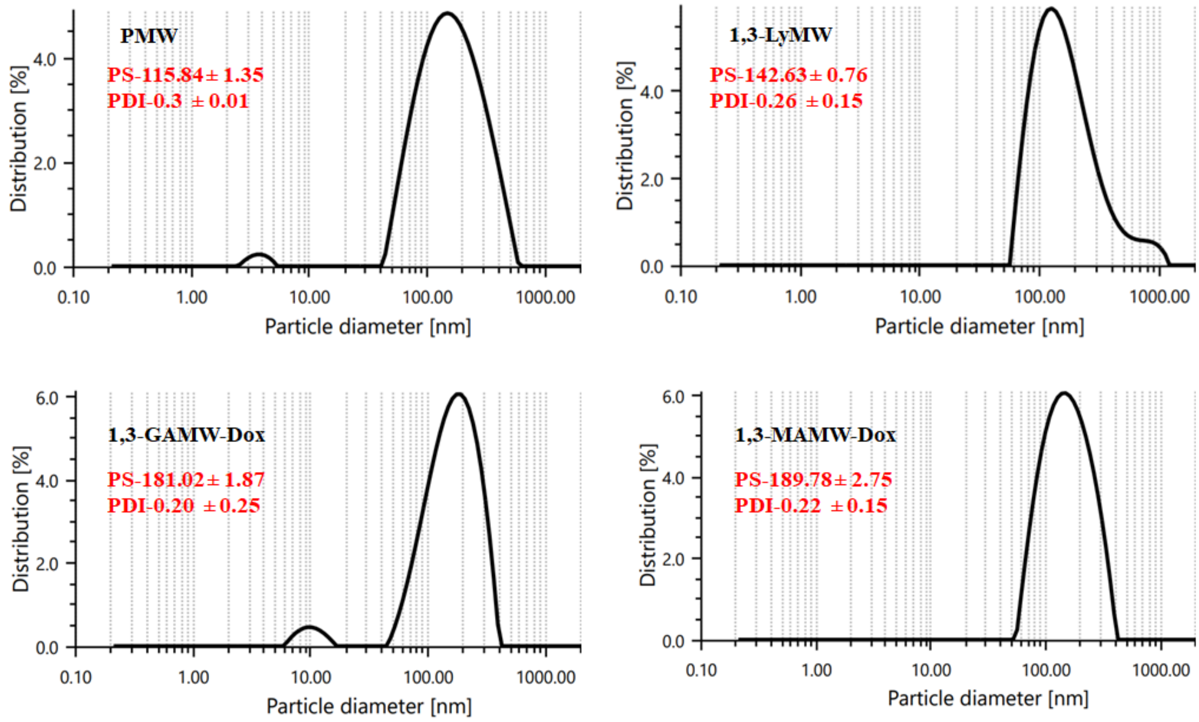

Fig. S6 *Particle size (PS) and polydispersive index (PDI) of PMW, 1,3-Lysine-MWCNTs, 1,3-Galactose-LyMWCNTs and 1,3-Mannose-LyMWCNTs*

Zeta potential studies

Dynamic light scattering was used to assess the surface charge of pristine and functionalized MWCNTs. The zeta potential results for PMW, 1,3-LyMW, 1,3-GAMW-Dox, and 1,3-MAMW-Dox were -20.7±0.88, 33.9±2.90, 48.06±0.95, and 45.13±1.89 mV, respectively, as shown in Table S1. Because of the presence of protonated amine groups as well as electron withdrawing groups, the surface charge of MWCNTs changed from negative to positive after conjugation of lysine and Dox [20].

Table S1: Different properties of MWCNTs formulations such as particle size, PDI, zeta potential and % Dox loading

Formulations	Particle Size (nm)	Polydispersity Index	Zeta Potential (mV)	Dox Loading (%)
**PMW**	115.84 ± 1.35	0.3 ± 0.01	-20.7 ± 0.88	87.35 ± 2.61
**1,3-LyMW**	142.63 ± 0.76	0.26 ± 0.15	33.9 ± 2.90	94.01 ± 0.32
**1,3-GAMW-Dox**	181.02 ± 1.87	0.20 ± 0.25	48.06 ± 0.95	96.89 ± 1.58
**1,3-MAMW-Dox**	189.78 ± 2.75	0.22 ± 0.15	45.13 ± 1.89	98.07 ± 2.34

Molecular Dynamics Simulations

Table S2 - Partial RESP Charges for the Lysine functionalization, obtained using the Gaussian 09 software, with HF/6-31G(d) basis set, and assigned using Antechamber.

Atom	Partial Charge
C CNT	0.006812
N1	-0.692294
N2	-0.451364
C1	0.086600
C2	0.051098
C3	-0.207954
C4	0.081663
C5	-0.078853
C6	0.181387
H1	0.055730
H2	0.078498
H3	0.055730
H4	0.393668
H5	0.093718
H6	0.093718
H7	0.003548
H8	0.003548
H9	0.043584
H10	0.043584
H11	0.058279
H12	0.058279
H13	0.342465
H14	0.342465
H15	0.342465

Table S3 - Partial RESP Charges for the Galactose functionalization, obtained using the Gaussian 09 software, with HF/6-31G(d) basis set, and assigned using Antechamber.

Atom	Partial Charge	Atom	Partial Charge
C CNT	-0.085548	H3	0.087711
N1	-0.755042	H4	0.062060
N2	-0.424451	H5	0.091490
O1	-0.644593	H6	0.072591
O2	-0.643332	H7	0.018909
O3	-0.670733	H8	0.055411
O4	-0.586291	H9	0.019882
O5	-0.705391	H10	0.019882
C1	0.072795	H11	0.411597
C2	0.205390	H12	0.396483
C3	0.227017	H13	0.431825
C4	0.191979	H14	0.347784
C5	0.258056	H15	0.429841
C6	0.154001	H16	0.364392
C7	0.182111	H17	0.039335
C8	0.140951	H18	0.039335
C9	-0.043834	H19	-0.018352
C10	0.081879	H20	-0.018352
C11	-0.000349	H21	0.010699
C12	0.091645	H22	0.010699
H1	0.087711	H23	0.034400
H2	0.095098	H24	0.034400

Table S4 - Partial RESP Charges for the Mannose functionalization, obtained using the Gaussian 09 software, with HF/6-31G(d) basis set, and assigned using Antechamber.

Atom	Partial Charge	Atom	Partial Charge
C CNT	0.011497	H3	0.196344
N1	0.096657	H4	0.098172
N2	-0.733183	H5	0.104224
O1	-0.642471	H6	0.069664
O2	-0.713139	H7	0.072242
O3	-0.692013	H8	0.154425
O4	-0.735664	H9	0.021473
O5	-0.673226	H10	0.021473
C1	0.015780	H11	0.384396
C2	0.428499	H12	0.401666
C3	0.292442	H13	0.401201
C4	-0.166816	H14	0.369186
C5	0.224917	H15	0.405033
C6	0.377206	H16	0.211004
C7	-0.553560	H17	-0.014730
C8	-0.485433	H18	-0.014730
C9	0.342356	H19	-0.011598
C10	-0.095063	H20	-0.011598
C11	-0.566208	H21	0.140052
C12	0.640735	H22	0.140052
H1	0.196344	H23	0.037018
H2	0.195396	H24	0.037018

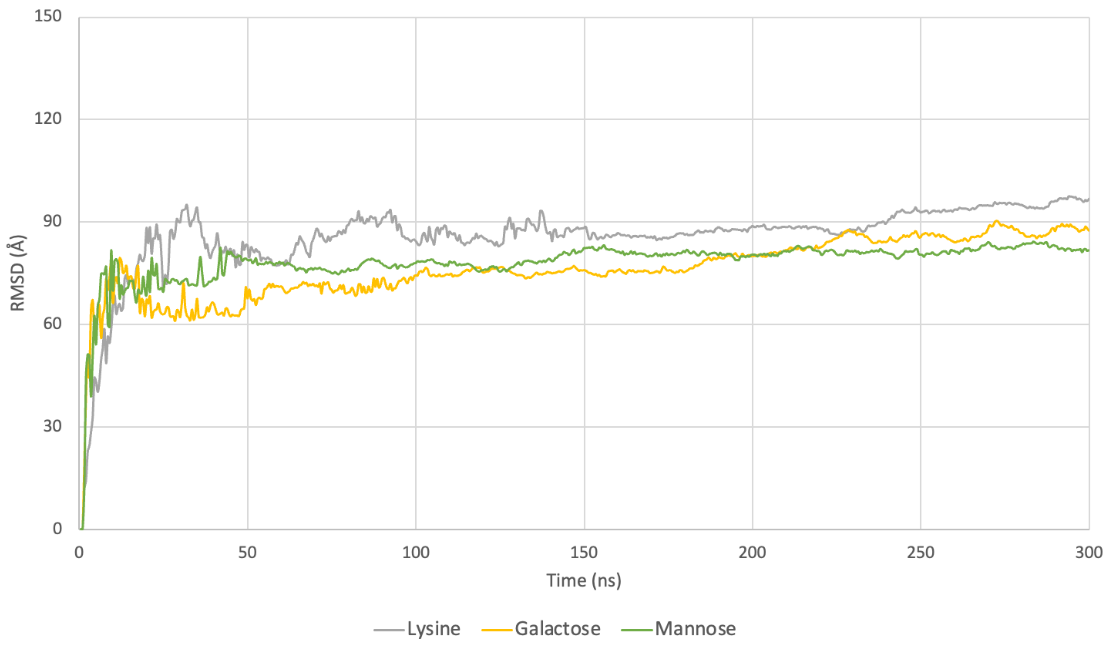

Fig. S7 *Average root mean square deviation for all the doxorubicin molecules, in simulations with neutral pH*

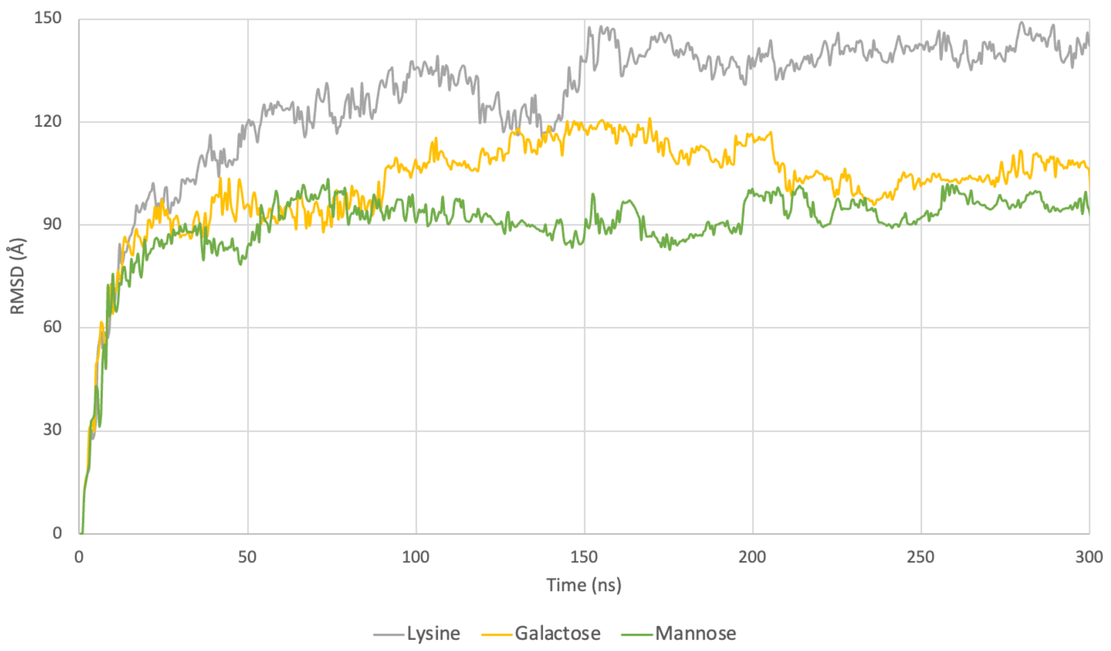

Fig. S8 *Average root mean square deviation for all the doxorubicin molecules, in simulations with acidic pH*

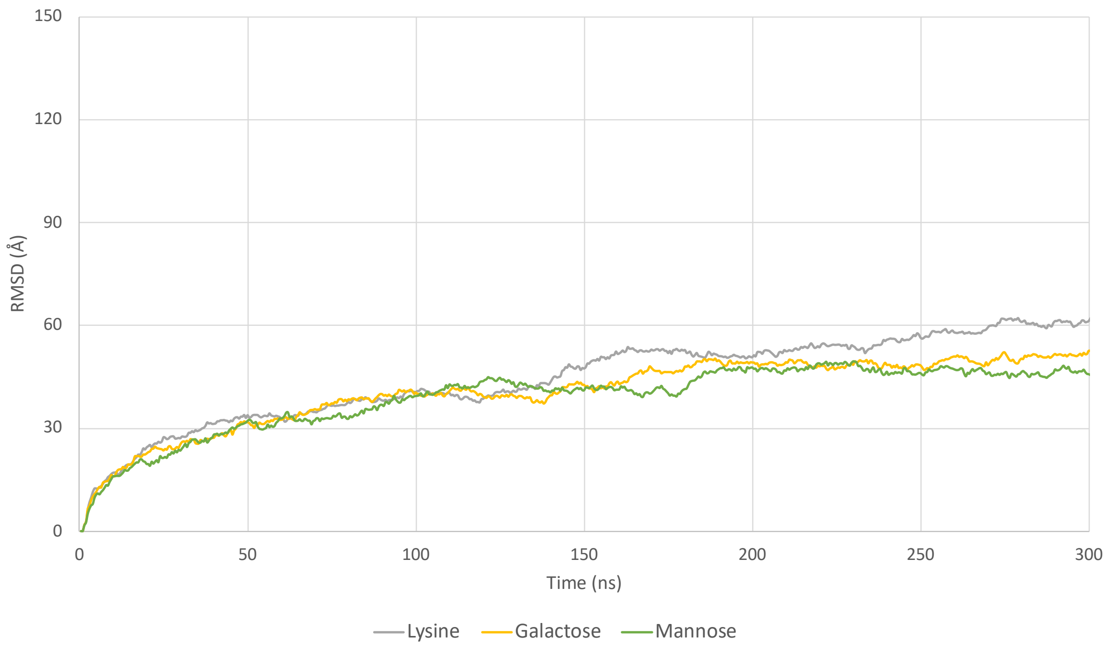

Fig. S9 *Average root mean square deviation for all the doxorubicin molecules, in simulations where the pH was changed from neutral to acidic*

References

[1]. Mulvey JJ, Feinberg E, Alidori S, McDevitt M, Heller D, Scheinberg D. Synthesis, pharmacokinetics, and biological use of lysine-modified single-walled carbon nanotubes. Int J Nanomed 2014;9:4245–4255. https://doi.org/10.2147/IJN.S66050

[2]. Jain AK, Dubey V, Mehra NK, Lodhi N, Nahar M, Mishra DK, Jain N. Carbohydrate-conjugated multiwalled carbon nanotubes: development and characterization. Nanomed 2009;5(4):432–442. https://doi.org/10.1016/j.nano.2009.03.001

[3]. Pruthi J, Mehra NK, Jain NK. Macrophages targeting of amphotericin B through mannosylated multiwalled carbon nanotubes. J Drug Target 2012;20(7):593–604. https://doi.org/10.3109/1061186X.2012.697168.

[4]. Thakur CK, Neupane R, Karthikeyan C, Ashby Jr, CR, Babu RJ, Boddu SHS, Tiwari AK, Moorthy NSHN. Lysinated multiwalled carbon nanotubes with carbohydrate ligands as an effective nanocarrier for targeted doxorubicin delivery to breast cancer cells. Molecules 2022;27:7461.

[5]. Garg NK, Singh B, Kushwah V, Tyagi RK, Sharma R, Jain S, Katare OP. The ligand (s) anchored lipobrid nanoconstruct mediated delivery of methotrexate : an effective approach in breast cancer therapeutics. Nanomed: Nanotechnol Biol Med 2016;12:2043–2060.

[6]. Pastorin G, Wu W, Wieckowski S, Briand JP, Kostarelos K, Prato M, Bianco A. Double functionalisation of carbon nanotubes for multimodal drug delivery. Chem Comm 2006;11:1182–1184. https://doi.org/10.1039/b516309a.

[7]. Kumar A, Rao MV, Menon SK. Photoinduced DNA cleavage by fullerene – lysine conjugate. Tetrahedron Lett 2009;50(47):6526–6530. https://doi.org/10.1016/j.tetlet.2009.09.027.

[8]. Iftime MM, Morariu S, Marin L. Salicyl-imine-chitosan hydrogels : Supramolecular architecturing as a crosslinking method toward multifunctional hydrogels. Carbohyd Polym 2017;165:39–50. https://doi.org/10.1016/j.carbpol.2017.02.027.

[9]. Marin L, Ailincai D, Morariu S, Tartau-Mititelu L. Development of biocompatible glycodynameric hydrogels joining two natural motifs by dynamic constitutional chemistry. Carbohyd Polym 2017;170:60–71. https://doi.org/10.1016/j.carbpol.2017.04.055.

[10]. Ansari SA, Satar R, Chibber S, Khan MJ. Enhanced stability of Kluyveromyces lactis beta galactosidase immobilized on glutaraldehyde modified multiwalled carbon nanotubes. J Mol Catal B-Enz 2013;97:258–263. https://doi.org/Doi 10.1016/J.Molcatb.2013.09.008.

[11]. Haroun A, Gospodinova Z, Krasteva N. Amino acid functionalization of multi-walled carbon nanotubes for enhanced apatite formation and biocompatibility. Nano Biomed Eng 2021;13:380–393. https://doi.org/10.5101/nbe.v13i4.

[12]. Mallakpour S, Behranvand V. Improved solubilization of multiwalled carbon nanotubes (MWCNTs) in water by surface functionalization with D-glucose and D-fructose : Properties comparison of functionalized MWCNTs/alanine-based poly (amide–imide) nanocomposites. High Performance Poly 2016;28(8):936–944. https://doi.org/10.1177/0954008315610395.

[13]. Delhaes P, Couzi M, Trinquecoste M, Dentzer J, Hamidou H, Vix-Guterl C. A comparison between Raman spectroscopy and surface characterizations of multiwall carbon nanotubes. Carbon

2006;44:3005–3013. https://doi.org/10.1016/j.carbon.2006.05.021.

[14]. Muda MR, Ramli MM, Mat Isa SS, Halin DSC, Talip LFA, Mazelan NS, Anhar NAM, Danial NA. Structural and morphological investigation for water-processed graphene oxide/single-walled carbon nanotubes hybrids. Mater Sci Eng 2017;209:2–8. https://doi.org/10.1088/1757-899X/209/1/012030

[15]. Gu L, Luo PG, Wang H, Meziani MJ, Lin Y, Veca LM, Cao L, Lu F, Wang X, Quinn RA, Wang W, Zhang P, Lacher S, Sun YP. Single-walled carbon nanotube as a unique scaffold for the multivalent display of sugars. Biomacromol 2008;9(9):2408–2418. https://doi.org/10.1021/bm800395e

[16]. Amiri A, Maghrebi M, Baniadam M, Heris SZ. One-pot, efficient functionalization of multi-walled carbon nanotubes with diamines by microwave method. Appl Surf Sci 2011;257(23):10261–10266. https://doi.org/10.1016/j.apsusc.2011.07.039.

[17]. de Sousa M, Martinez DST, Alves OL. Alternative mannosylation method for nanomaterials: application to oxidized debris-free multiwalled carbon nanotubes. J Nanoparticle Res 2016;18(6):143. https://doi.org/10.1007/s11051-016-3399-9.

[18]. Kumar NA, Bund A, Cho BG, Lim KT, Jeong YT. Novel amino-acid-based polymer/multi-walled carbon nanotube bio-nanocomposites: highly water dispersible carbon nanotubes decorated with gold nanoparticles. Nanotechnol 2009;20:225608. https://doi.org/10.1088/0957-4484/20/22/225608.

[19]. Raza K, Kumar D, Kiran C, Kumar M, Guru SK, Kumar P, Arora S, Sharma G, Bhushan S, Katare OP. Conjugation of docetaxel with multiwalled carbon nanotubes and codelivery with piperine: Implications on pharmacokinetic profile and anticancer activity. Mol Pharmaceut 2016;13(7):2423–2432. https://doi.org/10.1021/acs.molpharmaceut.6b00183.

[20]. Singhai NJ, Maheshwari R, Jain NK, Ramteke S. Chondroitin sulphate and α-tocopheryl succinate tethered multiwalled carbon nanotubes for dual-action therapy of triple-negative breast cancer. J Drug Del Sci Technol 2020;60:102080. https://doi.org/10.1016/j.jddst.2020.102080.
